# Thermogenic adipose tissue in older adults with obesity: a narrative review of mechanisms, brown fat resistance, and the translational relevance of exercise and nutrition

**DOI:** 10.3389/fnut.2026.1818342

**Published:** 2026-05-14

**Authors:** Fanchang Wang, Hongyang Qiao, Hongxin Zhou, Yi Zheng, Yuxin Ni, Xiaoming He

**Affiliations:** 1 The Second School of Clinical Medicine, Zhejiang Chinese Medical University, Hangzhou, China; 2 The First Affiliated Hospital of Wenzhou Medical University, Wenzhou, China; 3 The Third Clinical College of Shanxi University of Chinese Medicine, Taiyuan, China; 4 The Second Affiliated Hospital of Zhejiang Chinese Medical University, Hangzhou, China

**Keywords:** beige adipocytes, brown adipose tissue, brown fat resistance, exercise, nutrition

## Abstract

Population aging is accelerating, and obesity is becoming more common in older adults, creating a growing clinical burden. Brown adipose tissue (BAT), together with inducible beige adipocytes in white fat depots, supports adaptive thermogenesis through substrate oxidation and helps clear glucose and lipids from the circulation. Aging and obesity often coexist, reducing BAT volume and activity while lowering the browning capacity of white adipose tissue. Thermogenic responsiveness to cold exposure, exercise, and diet-related signals appears attenuated in this context. This pattern is often described as brown fat resistance. In real-life settings, it may make older adults’ responses to lifestyle interventions less consistent and less pronounced. This narrative review synthesizes evidence from animal models and human studies to delineate the principal mechanisms by which exercise and nutrition shape BAT and beige adipose biology. Relevant English-language articles published up to December 2025 were identified through PubMed, Scopus, and Web of Science, and screened according to their topical and methodological relevance. We also examine how age-related limits, including sarcopenia, chronic low-grade inflammation, weaker sympathetic and β-adrenergic signaling, and endocrine imbalance, raise the activation threshold and reduce thermogenic responses. Much mechanistic evidence comes from rodent studies or young, metabolically healthy populations, whereas human findings in obese older adults remain heterogeneous because of differences in endpoints, cold-stimulation protocols, phenotype characterization, and small sample sizes. Based on this evidence, we outline an integrated framework that links structured exercise with thermogenesis-supportive nutrition, while cautioning against overinterpretation of surrogate imaging readouts. The aim is to make thermogenic adipose activation more feasible in obese older adults by improving whole-body metabolic conditions and substrate handling, strengthening inter-organ communication, and increasing adipose tissue sensitivity to external triggers. We also highlight the need for future trials to use clear prespecified stratification suitable for older adults, and to establish standard safety checks and monitoring plans that support practical and individualized strategies targeting BAT and beige adipose tissue.

## Introduction

Global population aging is accelerating, and obesity is becoming more common (1, 2). Older adults now make up a growing share of people living with obesity, and the impact on cardiovascular and metabolic health in later life is becoming clearer (3). Excess adiposity worsens insulin resistance and dyslipidaemia and commonly coexists with age related loss of skeletal muscle mass and function, producing the clinical phenotype termed sarcopenic obesity (4, 5).

Adipose tissue remodeling is a critical, yet frequently overlooked, driver of metabolic decline with aging (6). Thermogenic adipocytes, including classical brown adipocytes in brown adipose tissue and inducible beige adipocytes within white adipose depots, dissipate energy as heat and contribute to systemic glucose and lipid disposal (7). In humans, brown adipose tissue persists into adulthood and is typically identified in the supraclavicular, cervical, mediastinal, and paravertebral regions (8–10). With advancing age, brown adipose tissue volume and activity generally decrease, whereas visceral white adipose tissue more readily expands and acquires an inflammatory phenotype with reduced metabolic competence (11). Landmark PET/CT studies published in 2009 demonstrated metabolically active brown adipose tissue in adults, overturning the prior assumption that it was restricted to infants and small mammals (9, 10). Subsequent work confirmed that both classical brown adipose tissue and recruitable beige adipocytes express uncoupling protein 1 (UCP1), are enriched in mitochondria, and, when activated, can increase energy expenditure and improve metabolic regulation (12). Collectively, these observations support thermogenic adipose tissue as a biologically plausible target for interventions aimed at obesity and related metabolic disorders. However, existing syntheses have largely treated the exercise, nutrition, and mechanistic BAT literatures separately, mostly drawing on studies in young, metabolically healthy individuals. The translational implications for obese older adults, whose BAT biology is shaped by aging, adiposity, comorbidity, and polypharmacy, therefore remain insufficiently examined. As a result, it is still unclear which candidate stimuli remain biologically plausible under age- and obesity-related constraints, and which are unlikely to succeed without prior improvement of the systemic milieu.

Most mechanistic insights into brown adipose tissue activation, white adipose tissue browning, and thermogenesis-promoting nutrients are derived from animal models and younger cohorts. Therefore, interpretation in obese older adults should emphasize endpoint context, phenotype differences, and feasibility constraints. Older individuals often differ in physiological reserve, cardiometabolic comorbidity burden, medication exposure, and body composition, which can modify thermogenic readouts and intervention responses. Age-associated systemic alterations may raise the activation threshold of thermogenic adipose tissue, which can make lifestyle effects more heterogeneous across older adults (13).

In this review, we integrate available evidence on exercise and nutrition interventions relevant to brown adipose tissue activation in older adults and argue that intervention development should be anchored in the central concept of age related brown fat resistance. For this review, age-related brown fat resistance refers to a tissue-level phenotype in obese older adults characterized, relative to metabolically matched younger individuals, by a higher threshold for thermogenic recruitment, weaker responses to physiological cues, and reduced durability of induced beige phenotypes after stimulus withdrawal. A fuller definition and its distinction from related constructs are presented in the section on age-related brown fat resistance. Using this core concept, the present review aims to inform intervention programs for obese older adults that are both biologically sound and clinically feasible. The present synthesis differs from earlier narrative reviews in three ways. First, it brings the exercise and nutrition literatures together within a single framework of age-related brown fat resistance, rather than treating them separately. Second, it makes explicit where findings from rodent studies or younger cohorts may not translate well to obese older adults. Third, it extends this mechanistic synthesis into a phenotype-informed, safety-aware intervention framework that prioritizes preparation of the systemic milieu over high-intensity or pharmacologic stimuli that may be poorly tolerated. Taken together, these features shift the focus from whether BAT can be activated in principle to the conditions under which such activation may be achievable and clinically meaningful in obese older adults.

## Methods

This narrative review was conducted with reference to the Scale for the Assessment of Narrative Review Articles (SANRA). PubMed/MEDLINE, Scopus, and Web of Science Core Collection were searched from database inception to 31 December 2025 for English-language, peer-reviewed publications. The search strategy combined terms related to thermogenic adipose tissue with terms related to aging or obesity, exercise, and nutrition, using AND between concept blocks and OR within each block. Searches were performed in title and abstract fields in PubMed, TITLE-ABS-KEY in Scopus, and topic fields in Web of Science. The database-specific search records, search-field settings, and Boolean concept blocks are summarized in the Supplementary Material 1 to improve methodological transparency, while recognizing that this article is a structured narrative review rather than a systematic review or meta-analysis.

Eligible records included peer-reviewed original studies and relevant reviews examining exercise-related, nutrition-related, or age-related effects on brown or beige adipose tissue. Exclusion criteria were abstracts, editorials, letters, preprints, duplicate records, studies without a clear focus on brown or beige adipose tissue, studies limited to fetal or neonatal populations, and records for which the full text was not accessible.

After deduplication, two authors independently screened titles, abstracts, and keywords, followed by detailed relevance assessment of potentially relevant records, including full-text review when necessary. Disagreements were resolved through discussion or, when needed, consultation with a third author. Reference lists of included articles and key reviews were also screened manually. Given the methodological heterogeneity of the included literature, findings were synthesized narratively across four themes: thermogenic adipose biology, age-related brown fat resistance, exercise-mediated modulation, and the effects of nutrition or bioactive compounds. The selection process is summarized in a simplified PRISMA-style flow diagram in Supplementary Figure 1.

**FIGURE 1:**
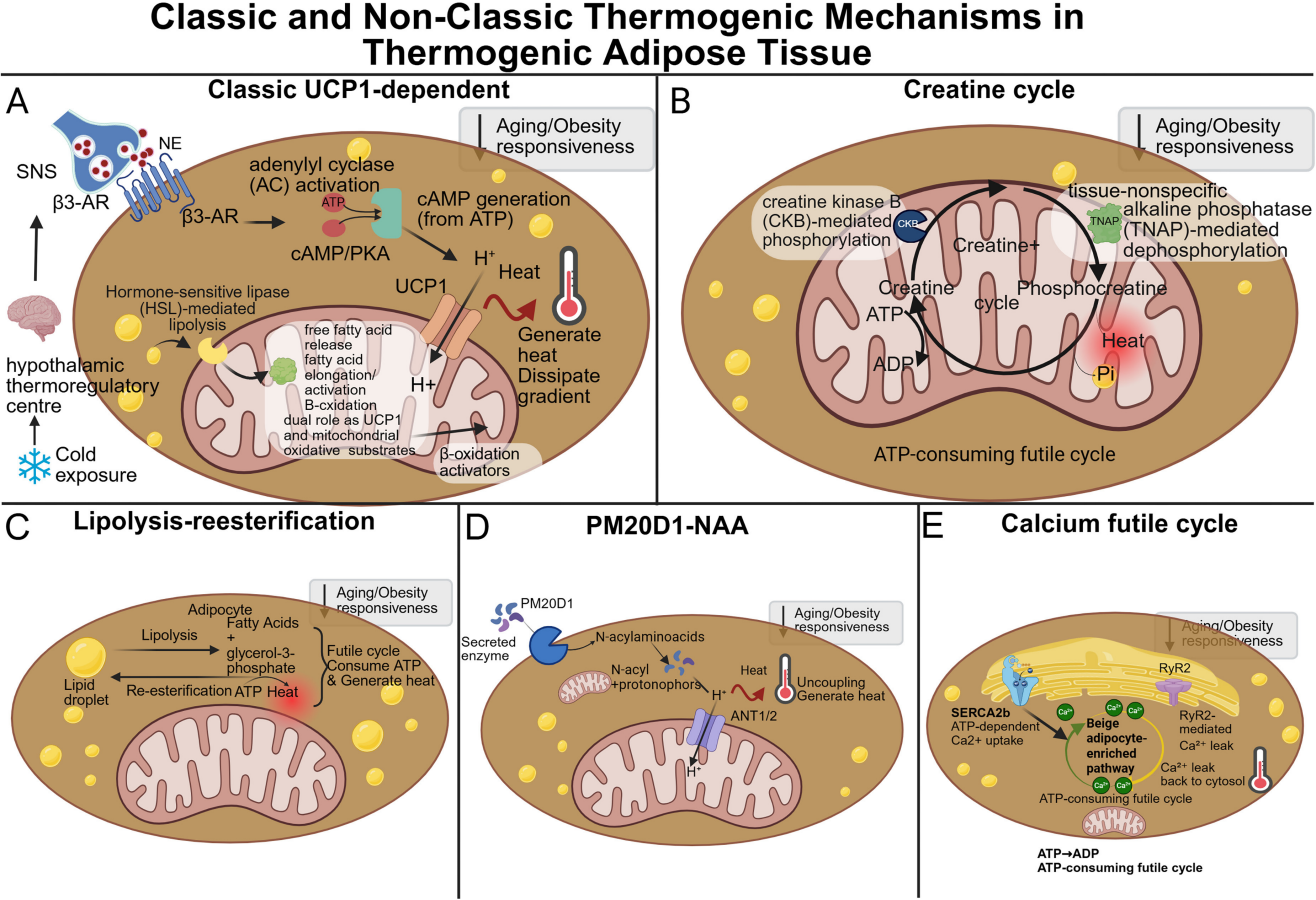
Classic and non-classic thermogenic mechanisms in thermogenic adipose tissue. **(A)** In classic UCP1-dependent thermogenesis, cold exposure activates the hypothalamic thermoregulatory center and sympathetic nervous system, leading to norepinephrine release and β3-adrenergic receptor activation in brown adipocytes. This stimulates adenylyl cyclase, promotes cAMP generation from ATP, activates the cAMP/PKA pathway, and induces hormone-sensitive lipase-mediated lipolysis. Released fatty acids serve as mitochondrial oxidative substrates and activate UCP1-dependent proton leak, thereby dissipating the proton gradient as heat. **(B)** The creatine cycle represents a UCP1-independent ATP-consuming futile cycle. Creatine kinase B (CKB) mediates creatine phosphorylation, whereas tissue-non-specific alkaline phosphatase (TNAP) promotes phosphocreatine dephosphorylation, resulting in continuous ATP turnover and heat generation. **(C)** Lipolysis and re-esterification form another futile cycle in which fatty acids released from lipid droplets are re-esterified back into triglycerides, consuming ATP and contributing to energy dissipation as heat. **(D)** The PM20D1-N-acyl amino acid pathway generates endogenous uncoupling molecules that can increase mitochondrial proton conductance and promote heat production independently of UCP1 to some extent. **(E)** The calcium futile cycle, enriched in beige adipocytes, involves ATP-dependent Ca^2+^ uptake by SERCA2b and RyR2-mediated Ca^2+^ leak back into the cytosol, thereby sustaining ATP consumption and heat production. Grey boxes indicate that ageing and obesity may attenuate the responsiveness of each thermogenic pathway. Created in BioRender. Qiao, H. (2026) https://BioRender.com/tudwywh.

**FIGURE 2:**
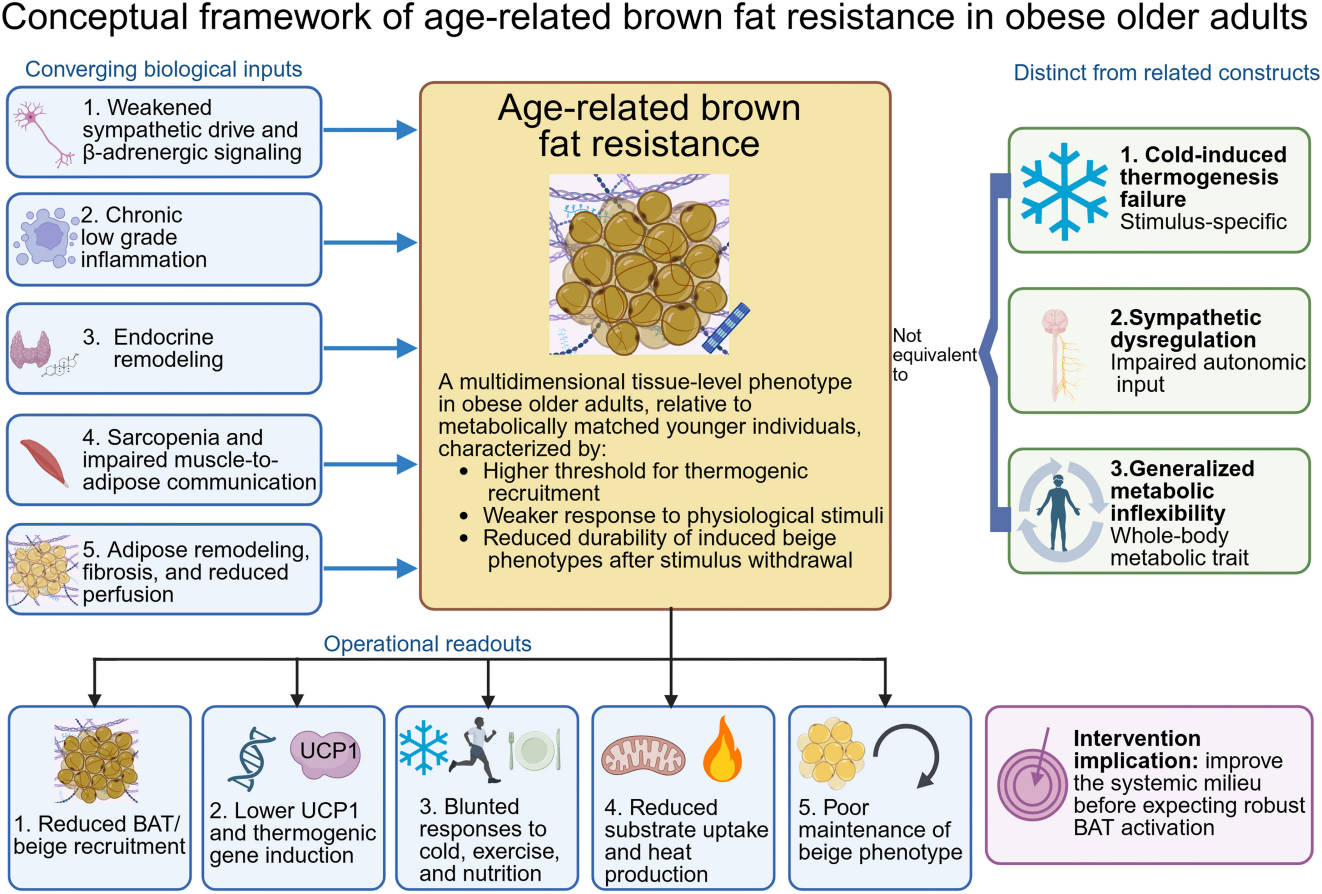
Conceptual framework of age-related brown fat resistance in obese older adults. This figure presents age-related brown fat resistance as a multidimensional tissue-level phenotype in obese older adults, relative to metabolically matched younger individuals. The central construct is characterized by a higher threshold for thermogenic recruitment, weaker responses to physiological stimuli, and reduced durability of induced beige phenotypes after stimulus withdrawal. The left side summarizes converging biological inputs, including weakened sympathetic drive and β-adrenergic signaling, chronic low-grade inflammation, endocrine remodeling, sarcopenia with impaired muscle-to-adipose communication, and adipose remodeling with fibrosis and reduced perfusion. The lower panel lists operational readouts, including reduced BAT or beige recruitment, lower UCP1 and thermogenic gene induction, blunted responses to cold, exercise, and nutrition, reduced substrate uptake and heat production, and poor maintenance of the beige phenotype. The right side distinguishes this construct from cold-induced thermogenesis failure, sympathetic dysregulation, and generalized metabolic inflexibility. The framework suggests that improving the systemic metabolic and inflammatory milieu may be necessary before expecting robust BAT activation in obese older adults. Created in BioRender. Qiao, H. (2026) https://BioRender.com/5945rd7.

**FIGURE 3:**
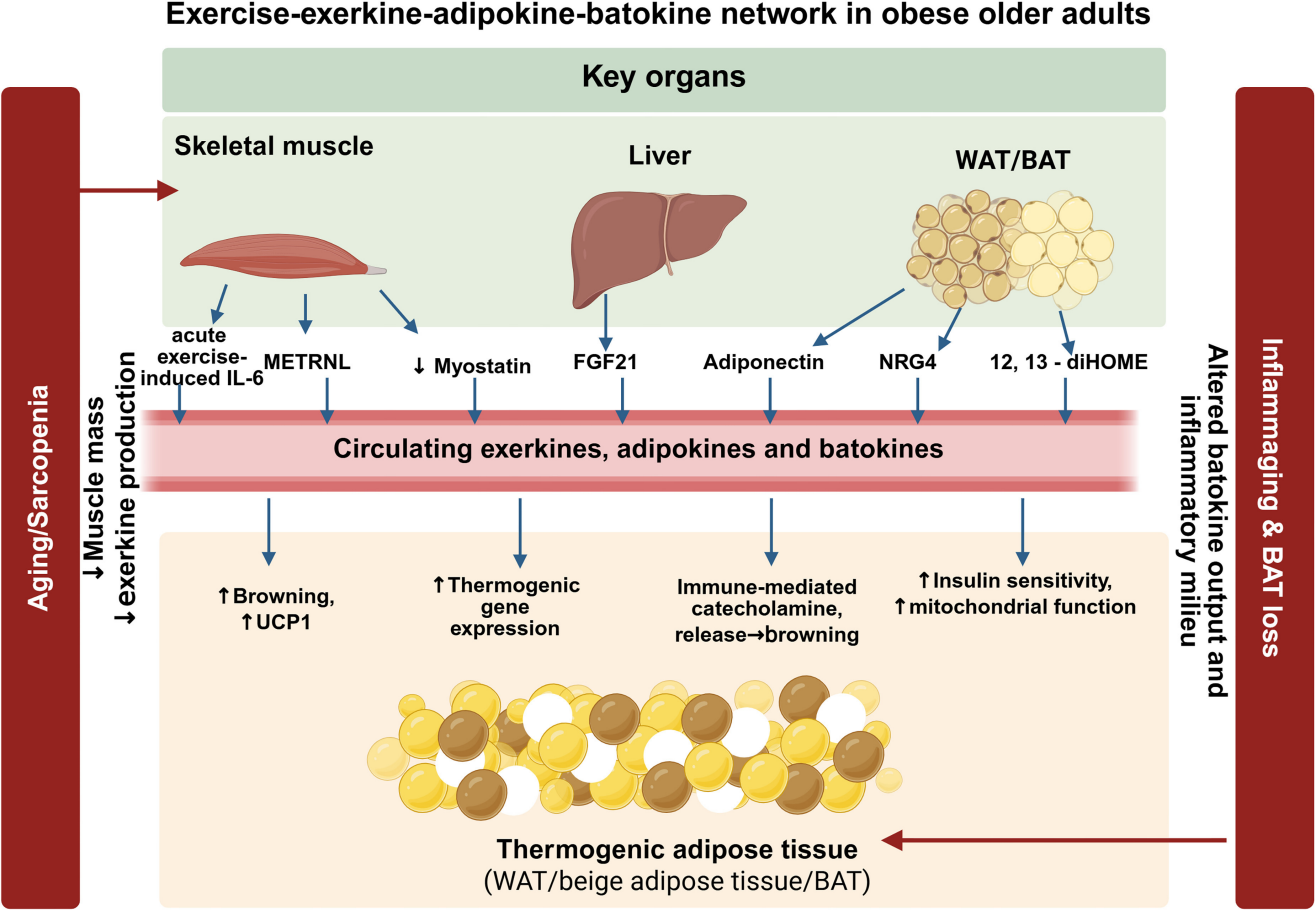
Exercise-exerkine-adipokine-batokine network in obese older adults. This Figure illustrates how exercise may influence thermogenic adipose tissue through inter-organ communication among skeletal muscle, liver, white adipose tissue, beige adipocytes, and brown adipose tissue. Skeletal muscle contraction can induce exercise-responsive mediators such as acute exercise-induced IL-6 and METRNL, while reducing adverse myostatin signaling. The liver and adipose tissues contribute additional circulating mediators, including FGF21, adiponectin, NRG4, and 12,13-diHOME. Together, these exerkines, adipokines, and batokines may support browning, UCP1-related thermogenic gene expression, catecholamine-linked remodeling, insulin sensitivity, and mitochondrial function. In obese older adults, sarcopenia, reduced muscle mass, altered batokine output, inflammaging, and BAT loss may weaken this endocrine network and contribute to impaired thermogenic responsiveness. Created in BioRender. Qiao, H. (2026) https://BioRender.com/hwhcbso.

**FIGURE 4:**
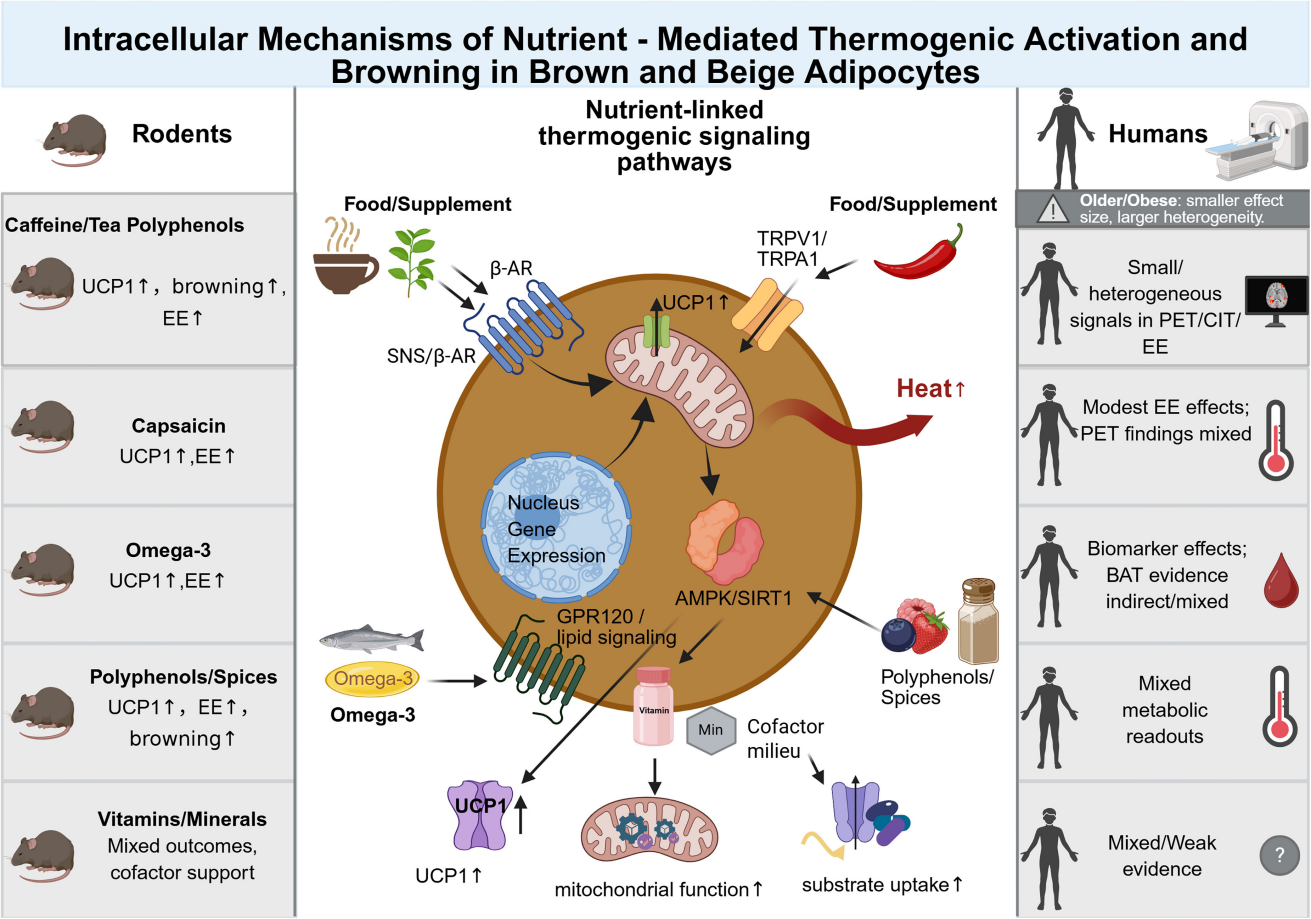
Intracellular Mechanisms of nutrient-mediated thermogenic activation and browning in brown and beige adipocytes. This figure summarizes candidate thermogenic nutrients and dietary bioactive compounds according to their main pathway nodes and compares the strength of evidence between rodent and human studies. The left column summarizes typical rodent findings, in which caffeine or tea polyphenols, capsaicin, omega-3 fatty acids, polyphenols or spices, and selected vitamins or minerals have been associated with changes in UCP1 expression, browning markers, energy expenditure, or mitochondrial support. The central panel illustrates putative nutrient-linked signaling pathways, including sympathetic and β-adrenergic signaling, TRPV1/TRPA1 activation, GPR120 or lipid-mediated signaling, AMPK/SIRT1-related mitochondrial adaptation, and cofactor support for substrate uptake and oxidative metabolism. The right column highlights that human evidence is generally smaller, more heterogeneous, and often based on surrogate readouts such as PET uptake, cold-induced thermogenesis, energy expenditure, circulating biomarkers, or metabolic endpoints. In obese older adults, these nutrients should therefore be interpreted as adjunctive modulators of a permissive metabolic milieu rather than stand-alone thermogenic interventions. Created in BioRender. Qiao, H. (2026) https://BioRender.com/5cyedzb.

**FIGURE 5:**
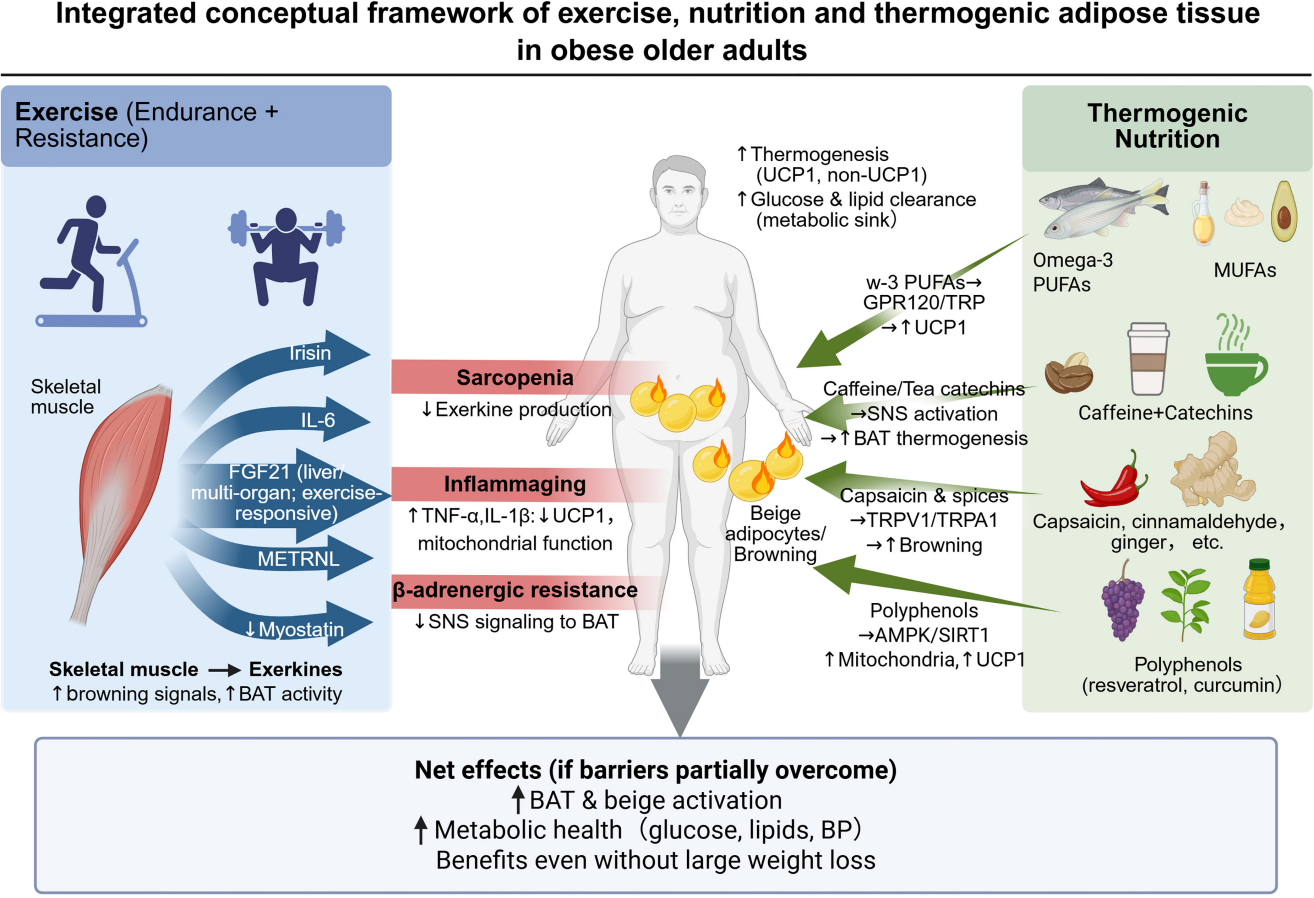
Integrated conceptual framework of exercise, nutrition, and thermogenic adipose tissue in obese older adults. This figure integrates exercise and nutrition as complementary strategies for improving the systemic and tissue-level conditions required for thermogenic adipose activation in obese older adults. Endurance and resistance exercise may preserve skeletal muscle mass and promote exercise-responsive signals, including irisin, acute exercise-induced IL-6, FGF21, METRNL, and reduced myostatin signaling. Thermogenic nutrition may provide additional pathway-specific inputs, including omega-3 PUFA-related GPR120 or lipid signaling, caffeine and tea catechin-related sympathetic activation, capsaicin and spice-related TRPV1/TRPA1 signaling, and polyphenol-related AMPK/SIRT1-mediated mitochondrial adaptation. These potential effects are constrained by sarcopenia, inflammaging, and β-adrenergic resistance, which may reduce exerkine production, impair mitochondrial function, and weaken sympathetic signaling to BAT. If these barriers are partially overcome, the expected net effects may include greater BAT and beige adipose activation, improved glucose and lipid clearance, better cardiometabolic health, and clinically meaningful benefit even without large weight loss. Created in BioRender. Qiao, H. (2026) https://BioRender.com/yjw6pia.

Figure preparation. All schematic figures (Figures 1–5) were created with BioRender (BioRender.com; Science Suite Inc., Toronto, Canada) under a BioRender Academic Publication License granted to H.Q., which permits publication in academic journals and sublicensing under open-access models such as CC-BY 4.0, consistent with the open-access policy of Frontiers in Nutrition. The corresponding citation URLs are provided in the legend of each figure, and signed Confirmation of Publication and Licensing Rights documents have been provided to the Editorial Office.

## Thermogenic adipose tissue: brown and beige fat

### Brown adipose tissue: canonical and non-canonical thermogenic pathways

Brown adipose tissue (BAT) is a principal adaptive thermogenic organ that contributes to thermoregulation and energy homeostasis by increasing substrate oxidation and converting chemical energy into heat (14). BAT is richly vascularized and receives dense sympathetic innervation (15). Brown adipocytes characteristically contain multilocular lipid droplets and abundant mitochondria, with uncoupling protein 1 (UCP1) localized to the inner mitochondrial membrane (16).

In addition to heat production, BAT activation promotes the clearance of circulating fatty acids and glucose, thereby modulating systemic lipid and glycemic homeostasis (17). Because human investigations of thermogenic adipose responses largely depend on imaging based readouts and surrogate physiological measures, Box 1 summarizes the interpretation of these endpoints and the major sources of between study variability.

BOX 1 **Interpreting human evidence on thermogenic adipose tissue (BAT/TAT)**

Endpoints

- 18F-FDG PET/CT uptake is a proxy of glucose utilization and BAT detectability under specific conditions; it does not always translate into quantified heat production and is influenced by insulin/feeding state and skeletal muscle glucose uptake.
- Fatty-acid tracers/oxidative metrics more closely reflect substrate utilization, but protocols are heterogeneous and less common in older cohorts.
- Indirect calorimetry and skin temperature are relatively non-specific and can be influenced by ambient temperature, clothing, and peripheral blood flow.
- Circulating ‘brown/beige’ factors are often correlational or acute signals and, on their own, are insufficient to confirm sustained tissue thermogenesis without complementary endpoints.
- Protocol Heterogeneity
- Ambient temperature, season, cold-stimulation dose/duration, and shivering control determine detectability and effect size.
- Phenotype/medication confounding
- Adiposity, insulin resistance/diabetes, thyroid status, inflammation, and perfusion modify BAT readouts.
- β-blockers and other drugs affecting sympathetic tone or metabolism may attenuate BAT responsiveness, which is common in older adults.
- Depot Biology
- Human depots exhibit heterogeneity; aging/obesity-associated remodeling diminishes recruitable capacity and imaging signal.
- Interpretation hierarchy
- In obese older adults, prioritize body composition (muscle preservation), insulin sensitivity, cardiometabolic markers, and function over BAT surrogates.
- Minimal reporting checklist
- Cold/ambient temperature + shivering control; season.
- Fasting/feeding status; recent physical activity; sleep/caffeine intake control.
- Key phenotype/comorbidities; medication background.
- Endpoint type + limitations; safety/tolerability.

Canonical BAT thermogenesis is initiated predominantly by cold triggered sympathetic outflow (18). Upon cold exposure, sympathetic nerve terminals innervating BAT release norepinephrine (NE), which binds β3-adrenergic receptors on brown adipocytes. Receptor activation engages Gαs-coupled signaling to stimulate adenylate cyclase, which catalyzes the conversion of ATP to cyclic AMP (cAMP); the resulting elevation of intracellular cAMP activates protein kinase A (PKA), which in turn stimulates lipolysis and upregulates thermogenic gene programs (19). PKA-driven lipolysis of intracellular triglycerides releases free fatty acids (FFAs); long-chain FFAs are further modified by adipocyte-expressed fatty-acid elongases and, upon reaching the mitochondrial inner membrane, function both as mitochondrial oxidative substrates and as activators of UCP1 dependent proton conductance across the inner mitochondrial membrane (20). By increasing proton leak, UCP1 uncouples electron transport from ATP synthesis and dissipates the electrochemical gradient as heat (21). In aggregate, fatty acid oxidation together with UCP1 mediated uncoupling constitutes the core UCP1 dependent thermogenic mechanism in BAT.

Available human evidence indicates that BAT detectability and cold-activated BAT activity decline with advancing age and greater adiposity, and preliminary cold-exposure PET data further suggest lower BAT uptake in older than in younger men; accordingly, obese older adults would be expected to exhibit a blunted BAT response to cold exposure (22–25). This attenuated responsiveness is linked not only to lower detectable BAT volume but also to functional deficits, including a higher activation threshold, reduced tissue perfusion, and compromised mitochondrial performance (11). Structural loss and functional impairment often develop at the same time, so imaging-based estimates may not fully capture true physiological capacity. These limits together can restrict BAT recruitment in obese older adults and reduce its role in energy balance and metabolic homeostasis. As a result, approaches that depend on a strong sympathetic drive to activate BAT may show less consistent efficacy in this population. This pattern reflects a recurring inconsistency in the field. Rodent cold-exposure studies typically show robust BAT recruitment, increased UCP1 expression, and higher energy expenditure, whereas human studies, particularly in obese or older adults, often report small, heterogeneous, or null effects on ^18^F-FDG-defined BAT activity. The gap likely reflects both interspecies differences and the fact that rodent experiments are usually conducted in young, lean animals under short, intense cold challenges, conditions far removed from the aging, polypharmacy-exposed, and chronically inflamed background of obese older adults. Rodent findings should therefore be treated as hypothesis-generating, and human data should remain the main basis for clinical interpretation. Box 2 expands on these translational considerations, and Table 1 summarizes the preclinical and clinical evidence base for the principal exercise and nutrition interventions discussed in the subsequent sections.

BOX 2 **Preclinical-to-clinical translation in obese older adults**

A recurring challenge in interpreting this field is that the mechanistic framework of thermogenic adipose tissue has been established largely in young, lean rodents under short, intense cold challenges below thermoneutrality, whereas the clinical population of interest is older, obese, exposed to polypharmacy, and chronically inflamed, and is usually studied under near-thermoneutral conditions. Three specific translational gaps therefore require caution: (i) the short, intense cold exposures used in preclinical work rarely match the milder, more variable exposures that are tolerable in older adults, so effect sizes cannot be assumed to scale directly; (ii) preclinical studies are dominated by UCP1-related readouts, whereas human studies more often rely on ^1^8F-FDG uptake, a surrogate of glucose uptake that does not always correspond to UCP1 expression; and (iii) most cited preclinical studies are conducted in young, lean animals, so findings have to be extrapolated to the older, obese, polypharmacy-exposed phenotype of the target clinical population. Throughout this review, preclinical findings are therefore treated as hypothesis-generating, and statements about clinical applicability are anchored, where possible, in human evidence. When only animal data are available, this is stated explicitly, and Table 1 summarizes the evidence base for the principal interventions discussed.

**TABLE 1:** Preclinical versus clinical evidence for the principal lifestyle, nutritional, and pharmacological-adjunct interventions discussed, with recommendations for obese older adults.

Intervention	Preclinical (rodent) evidence	Human evidence	Recommendation in obese older adults
Aerobic exercise	In rodent models, endurance training has been reported to increase adipocyte precursor populations in BAT; UCP1-related findings are not uniform across studies.	RCTs show improved fitness and body composition; FDG-PET BAT signals often null in young healthy adults; data in obese older adults are limited.	Core intervention. Benefit should be judged from cardiometabolic and functional endpoints rather than BAT imaging.
Resistance training	Not emphasized as a distinct preclinical BAT literature in this review.	Most established clinical value is lean mass preservation; BAT imaging evidence is mixed.	Core intervention, particularly for sarcopenic obesity. Thermogenic benefit is indirect.
Caffeine	Mechanism: adenosine receptor blockade and increased sympathetic signaling.	Short-term studies show small rises in resting energy use and modest increases in supraclavicular skin temperature.	Optional adjunct. Careful safety checks in older adults (sleep, anxiety, blood pressure).
Capsaicin/TRPV1 agonists	Mechanism: TRPV1 channel activation with engagement of sympathetic-related signaling.	Modest effects on BMI, body weight, and waist circumference in meta-analysis of RCTs in adults with overweight or obesity.	Adjunctive dietary component; effects are modest and tolerance should be considered.
Resveratrol	Multiple rodent studies associate resveratrol with increased adipose oxidative capacity and UCP1-related pathway upregulation.	Small human studies report thermogenic gene-expression changes, whereas weight and metabolic outcomes remain inconsistent.	Not recommended as routine intervention; bioavailability limits translation.
Curcumin	Browning markers and AMPK activation reported in rodents.	Small RCTs show modest weight or glycemic effects; direct human BAT evidence lacking.	Evidence that curcumin produces sustained thermogenic benefit in humans remains preliminary.
Omega-3 PUFA (EPA/DHA)	Upregulates UCP1-related markers and mitochondrial pathways in rodents.	Most consistent effects at the population level are on cardiometabolic biomarkers (triglycerides, inflammation); effects on BAT are indirect.	May be considered within a Mediterranean-style dietary pattern; ≥250 mg/day EPA + DHA is a commonly used nutritional target rather than a BAT-specific dose.
Vitamin D	Depot- and context-dependent thermogenic effects in rodents.	No clear association between 25(OH)D and BAT metabolism in recent healthy adult cohort.	Correct deficiency only; do not frame as thermogenic intervention.
Iron	Mouse studies link iron handling to BAT mitochondrial respiration; ferritin heavy-chain knockout improves adaptive thermogenesis.	Human TFRC expression has been associated with obesity-related adipose phenotypes and UCP1 expression; observational only.	Human evidence should be interpreted with caution; phenotype-specific effects likely across aging and obesity.
B vitamins/thiamine	*In vitro* and rodent data on thermogenic cofactor roles.	Emerging human adipose SLC19A3 data; deficiency common in bariatric cohorts.	Correct deficiency; no role for high-dose supplementation.
β3-adrenergic agonists (e.g., mirabegron)	β3-AR activation recapitulates cold-driven sympathetic signaling to BAT (mechanistic basis).	Modest, heterogeneous BAT activation in humans; cardiovascular safety signals reported.	Investigational. Use only with careful cardiovascular monitoring and phenotype-informed selection.

Pharmacological approaches aligned with canonical BAT activation typically target β3 adrenergic receptors to recapitulate cold driven sympathetic signaling (26). Available evidence suggests that β3 adrenergic agonists can modestly increase BAT activity and yield metabolic benefits (27, 28), yet reported effects in humans are heterogeneous (29). Several studies report cardiovascular safety signals (e.g., elevations in heart rate and blood pressure), which may necessitate careful participant selection and rigorous safety monitoring in obese older adults with cardiometabolic risk (30–32). Accordingly, evaluation in well-characterized cohorts with prespecified safety surveillance may be the most informative near-term pathway for this population.

As shown in Figure 1, in addition to UCP1 dependent uncoupling, brown and beige adipose tissue have been reported to engage multiple UCP1 independent routes of energy dissipation (33). These mechanisms typically operate through elevated ATP demand or inefficient substrate cycling and may partially compensate when UCP1 mediated thermogenesis is constrained (34). Four illustrative pathways are frequently discussed. First, the creatine linked phosphocreatine cycle can increase ATP turnover and thereby promote heat production (35). Seminal work from the Kazak group has defined the molecular basis of this futile cycle: mitochondrial creatine kinase B (CKB) utilizes ATP to phosphorylate creatine to phosphocreatine (36), while mitochondrially localized tissue-non-specific alkaline phosphatase (TNAP) hydrolyzes phosphocreatine back to creatine and inorganic phosphate (37). The coordinated, opposing activity of CKB and TNAP drives continuous ADP regeneration that fuels thermogenic respiration in the absence of net chemical work. Preclinical studies associate this pathway with improved cold tolerance and higher energy expenditure, whereas findings in humans remain variable (38). Second, a futile lipid cycle, characterized by simultaneous lipolysis and re esterification, consumes ATP continuously and dissipates energy as heat (39), and several experimental models suggest that this process may be upregulated when UCP1 capacity is limited (40, 41). Third, the PM20D1 associated N acyl amino acid pathway generates bioactive lipids that act as endogenous uncouplers, increasing proton conductance and supporting thermogenesis independently of UCP1 to a certain extent (42). Fourth, as originally revealed by the Kajimura group, a calcium futile cycle operates prominently in beige adipocytes: SERCA2b actively imports Ca^2+^ into the endoplasmic/sarcoplasmic reticulum at the expense of ATP hydrolysis, while Ca^2+^ is simultaneously released back into the cytosol via ryanodine receptor 2 (RyR2), generating a continuous ATP-consuming Ca^2+^ cycle that produces heat and contributes to systemic glucose homeostasis even when UCP1 is absent (43). A recent follow-up study from the same group identified the ER-membrane peptide C4orf3 (also known as another-regulin, ALN) as a molecular determinant of this process; C4orf3/ALN uncouples SERCA2b-mediated Ca^2+^ transport from its ATP hydrolysis, rendering the SERCA2b–C4orf3 complex exothermic, and its genetic loss diminishes adipose thermogenesis and increases adiposity in mice (44). Current human studies have not yet established the relative contribution of these non-canonical pathways, particularly in obese older adults. Mechanistic studies using standardized endpoints would help clarify their clinical relevance for metabolic outcomes.

Accordingly, in obese older adults, a pragmatic strategy is to prioritize sustained exercise and dietary modification rather than depending on high intensity, short lived stimuli to elicit transient BAT activation (45). Over time, these interventions can improve basal metabolic function and the local tissue milieu, which may increase adipose responsiveness to physiologic cues and support more consistent, durable metabolic benefits.

### Adipose tissue browning, whitening, and loss of plasticity

In addition to classical brown adipose tissue, white adipose tissue in adult humans and rodents can acquire UCP1 positive beige adipocytes in response to specific stimuli, thereby gaining inducible thermogenic capacity (46, 47). Beige adipocytes exhibit features intermediate between white and brown fat, and their lipid droplet architecture can shift between multilocular and unilocular patterns as environmental conditions change (48). This responsiveness indicates that browning is not a fixed conversion, but a reversible and dynamic process that can regress after stimulus withdrawal (49).

Two principal cellular sources have been proposed. Their relative contribution appears to vary by depot, and much of the mechanistic evidence derives from rodent models (50). Beige adipocytes may arise from precursor populations within white adipose depots, and under browning stimuli such as cold exposure or β3-adrenergic stimulation, adipocytes within these depots may acquire beige-like features (51, 52). In subcutaneous adipose tissue, particularly in preclinical models, prolonged cold exposure or β3-adrenergic stimulation can increase beige adipocyte recruitment and thermogenic activation, and this phenotype can lose thermogenic gene expression and multilocular morphology and shift toward a less thermogenic state, including a masked beige-like state described in rodent studies, once the stimulus is removed (51, 52). These observations indicate that maintenance of the beige phenotype depends on continued stimulation and that this plasticity may be especially vulnerable to aging- and obesity-related constraints.

Beige adipocyte induction depends on several regulatory inputs that act together, rather than one single stepwise pathway (50). Sympathetic signaling plays a major upstream role, while endocrine factors and nuclear receptor driven gene programs can also turn on the browning machinery (53). Exercise is one modifiable factor, and it may support white adipose browning through muscle to adipose communication (54). Available evidence suggests that some myokines can raise UCP1 expression and increase mitochondrial gene networks (55). The variation across human studies likely reflects differences in design, stimulus strength, tissue sampling, and outcome measures. Individual differences, depot specific adipose features, and prior exposure history also shape beige adipocyte recruitment and functional capacity (56, 57).

When these mechanisms are considered in humans, thermogenic adipose tissue must be viewed as a heterogeneous system. Adult human thermogenic adipose depots, particularly in the supraclavicular region, may contain overlapping features of classical brown and recruitable beige/brite adipocytes (58). In experimental and preclinical settings, beige cell recruitment during white fat browning is more commonly observed in subcutaneous depots, whereas visceral fat typically shows more limited browning capacity (59, 60). Adiposity-related adipose niche remodeling may further shape depot heterogeneity (61). Even when thermogenic adipose tissue is difficult to detect at baseline, prolonged cold acclimation can still lead to measurable metabolic activation, suggesting that many adults retain an inducible thermogenic reserve.

Human studies also lack shared criteria for defining brown and beige adipogenesis. Some studies focus on tissue based evidence, such as histology and gene signals like higher UCP1 and mitochondrial gene expression, while other studies infer activity from imaging based metabolic readouts (62, 63). These methods reflect different biological aspects, so differences in methods likely add to the mixed results seen across populations. A clear example of this definitional heterogeneity is the dissociation reported in several adult cohorts between UCP1 abundance in supraclavicular adipose biopsies and ^18^F-FDG uptake in the same depot. High UCP1 expression does not always coincide with increased glucose uptake under cold stimulation, and detectable ^18^F-FDG uptake may occur without clearly enriched UCP1 signal. Because these measures reflect different aspects of thermogenic biology, studies relying on only one readout may reach different conclusions about BAT activation. In obese older adults, this limitation is amplified by the low baseline detectability of thermogenic adipose tissue, which may partly account for conflicting results across otherwise comparable studies.

Beige adipocytes also rely more on continued external input to remain stable than classical brown adipose tissue. Cold exposure, β3 adrenergic agonists, and exercise can support beige adipocyte recruitment, yet this phenotype can fade over time, and de-browning may occur when these inputs stop (64–66). This instability matters in aging and obesity. Aging can lower baseline beige adipocyte levels and reduce the ability to recruit and maintain them during stimulation, while obesity related energy excess, low physical activity, and chronic metabolic stress can further push adipose depots toward a less thermogenic and less flexible state (67–69). In obese older adults, these combined effects often appear as higher activation thresholds and weaker durability of the beige phenotype, which limits the role of thermogenic adipose tissue in energy balance and metabolic homeostasis.

In summary, obese older adults often show a reduced ability to start and sustain white adipose browning, which is a key feature of brown fat resistance. The next sections use this framework to clarify when exercise and nutrition strategies are most likely to help, with the aim of guiding practical weight management approaches for obese older adults.

## Age-related brown fat resistance: mechanisms in obese older adults

In older adults, both the ability to recruit brown adipose tissue and the tendency of white adipose tissue to undergo browning generally decline with age, a pattern often described as brown fat resistance (70, 71). This impairment in thermogenic function likely reflects converging mechanisms, including weakened sympathetic signaling, chronic low-grade inflammation, reduced endocrine support, sarcopenia with impaired muscle–adipose communication, and structural remodeling of adipose tissue (72–74).

For this review, age-related brown fat resistance is defined as a multidimensional, tissue-level phenotype in obese older adults that, compared with metabolically matched younger individuals, shows a higher threshold for thermogenic recruitment, a weaker response to physiological stimuli, and less durable maintenance of induced beige phenotypes.

This construct is distinct from failure of cold-induced thermogenesis, which is stimulus-specific, from sympathetic dysregulation, which reflects impaired autonomic input, and from generalized metabolic inflexibility, which describes a whole-body metabolic trait rather than a thermogenic adipose phenotype. Instead, brown fat resistance refers to the integrated tissue-level consequence of concurrent decline in sympathetic drive, endocrine support, muscle-to-adipose signaling, and adipose niche integrity with aging and obesity. Figure 2 summarizes these inputs and their measurable readouts, and each is discussed below.

### Sympathetic drive and impaired signaling

Aging is associated with alterations in autonomic regulation that collectively reduce effective sympathetic input to thermogenic adipose tissue (75). Although basal sympathetic tone may rise as a compensatory response to weight gain or insulin resistance, β adrenergic receptor responsiveness and downstream signal transduction within adipose depots commonly decline. In parallel, local neural density and norepinephrine signaling efficiency in aged fat can be reduced (76–78). Consequently, greater sympathetic activity in obese older adults does not always yield proportionate increases in adipose thermogenesis.

These changes help explain why canonical stimuli, including cold exposure and catecholaminergic activation, often show limited capacity to sustain robust induction of UCP1 and related thermogenic gene programs in aged adipocytes. Attenuated norepinephrine signaling may also impair BAT perfusion and substrate delivery during cold challenge, further limiting the thermogenic response (79–81).

### Chronic low-grade inflammation

With advancing age, the adipose tissue microenvironment shifts toward a more pro inflammatory profile, characterized by increased immune cell infiltration and higher levels of mediators such as TNF α and IL 1β (82, 83). This inflammatory milieu can impair mitochondrial function and blunt the induction of thermogenic gene programs, including UCP1. In parallel, inflammation promotes fibrotic remodeling, which disrupts tissue architecture and makes the microenvironment less conducive to browning and sustained heat production. As a result, effective thermogenic activation often requires stronger or more prolonged stimuli.

Chronic low grade inflammation can blunt thermogenic gene induction, and cytokine dynamics should be interpreted in light of exposure context. The former more commonly reflects ongoing metabolic stress and is associated with constraints on thermogenesis, whereas the latter may function as a context dependent regulator of energy metabolism (84–86). Overall, persistent inflammation is generally associated with an inflammatory–fibrotic milieu that hinders initiation and maintenance of thermogenic transcriptional programs.

### Endocrine and hormonal environment changes

Age related endocrine remodeling may reduce the sensitivity of brown and beige adipose tissue to external stimuli. Lower thyroid hormone availability or diminished tissue responsiveness has been linked in some studies to impaired thermogenic capacity, consistent with the role of triiodothyronine in regulating UCP1 expression and promoting mitochondrial biogenesis (87, 88). Declining sex steroid levels may also contribute. For example, estrogen related variation has been associated with redistribution of adipose depots and differences in detectable thermogenic fat, although results across cohorts are not fully concordant (89–91).

Alterations in the growth hormone and insulin like growth factor 1 axis may further influence thermogenic potential indirectly by affecting skeletal muscle maintenance and overall metabolic status (92). In parallel, insulin resistance and compensatory hyperinsulinemia are more common in later life, and this metabolic context may reduce the efficiency with which thermogenic adipose tissue is recruited in response to physiologic cues (93). Taken together, attenuation of sympathetic and thyroid linked signaling, combined with broader hormonal shifts in aging, may generate an endocrine milieu that is less permissive for effective thermogenic fat activation.

### Sarcopenia and weakened muscle-fat communication

Aging is commonly accompanied by declines in skeletal muscle mass and function, which reduce resting energy expenditure and may attenuate exercise related metabolic signaling from muscle to adipose tissue. Experimental work suggests that exercise evoked myogenic mediators can participate in beige adipocyte recruitment and modulate thermogenic responses, yet much of the supporting evidence is currently limited to animal models and *in vitro* systems (94). In aging, particularly in the setting of sarcopenia and habitual inactivity, impaired muscle secretory output and weakened muscle-to-adipose signaling may hinder engagement of thermogenic programs within adipose depots, thereby contributing to brown fat resistance; however, direct evidence in older adults remains limited, and much of the mechanistic support derives from animal studies (95–97).

### Adipose tissue remodeling and fibrosis

With advancing age, adipose extracellular matrix remodeling becomes more prominent and is commonly accompanied by progressive fibrosis, reduced angiogenic capacity, and greater vulnerability to local hypoxia (98, 99). Increased collagen deposition and cross linking stiffen the matrix and reshape the mechanical microenvironment, which can impair beige adipocyte recruitment and survival and weaken maintenance of thermogenic transcriptional programs (100, 101). At the same time, diminished perfusion limits oxygen and nutrient delivery, exacerbating metabolic stress and cellular injury, intensifying inflammatory signaling, and promoting further fibrotic remodeling. Reduced capillary density additionally constrains the substrate and oxygen supply required for high rate oxidative metabolism, thereby making sustained thermogenic activation more difficult to achieve (102, 103).

In summary, these interacting constraints likely underlie the widely observed phenotype of brown fat resistance in obese older adults. Compared with younger individuals, recruitment of thermogenic adipose tissue in response to common triggers such as cold exposure, physical training, or diet based cues is typically blunted and shows greater interindividual heterogeneity. Accordingly, a key limitation in obese older adults is often not the lack of stimuli, but reduced tissue sensitivity and a diminished capacity to translate signals into a robust thermogenic program.

In this context, exercise and nutritional strategies may be more readily implementable. Interventions that reduce chronic inflammatory burden, improve microvascular perfusion and substrate handling, preserve skeletal muscle function, and support adrenergic signaling alongside mitochondrial adaptation may help create a more permissive physiological milieu for initiating and sustaining thermogenic activity.

Human evidence in obese older adults is frequently inferred from imaging based readouts and surrogate physiological measures, and protocol differences in temperature control, stimulation dose, and participant phenotype can produce divergent conclusions. Future studies should prioritize standardized reporting of ambient and cold exposure conditions, prespecified subgroup stratification, and mechanistic endpoints that better link thermogenic biology to clinically meaningful metabolic and functional outcomes.

## Exercise and thermogenic adipose tissue

Direct intervention evidence on exercise-induced thermogenic adipose adaptations in obese older adults remains limited; accordingly, the following discussion integrates available human findings with mechanistic and translational evidence from broader adult and preclinical literature. An overview of the exercise–exerkine–adipokine–batokine network discussed in this section is provided in Figure 3. Regular physical activity plays a key role in healthy aging and supports body composition, cardiovascular risk factors, and insulin sensitivity (104–106). Skeletal muscle contraction raises energy use and heat production (107), which may briefly lower the need for brown adipose tissue mediated heat generation. At the same time, regular exercise activates signaling pathways that can help maintain the longer term function of both brown and beige adipose tissue (108, 109).

Even with these mechanistic considerations, reported exercise effects on human BAT remain variable across cohorts and protocols. In obese older adults, exercise most often leads to better body composition, improved metabolic control, and stronger physical function. Whether BAT imaging signals or other surrogate indices change reproducibly with exercise should be interpreted in light of Box 1, with careful attention to endpoint limitations and variability in study protocols and experimental conditions.

Three factors may underlie these heterogeneous findings: age-related biological changes, differences in metabolic status, and methodological variation across studies. Declines in β3-adrenergic signaling, myokine output, and mitochondrial biogenesis may raise the threshold for thermogenic remodeling (110–112), while inflammation, insulin resistance, and visceral adiposity may suppress thermogenic programs, including UCP1-related pathways, and may impair PGC-1α-associated mitochondrial regulation (62, 113). At the same time, differences in imaging modality, cold-acclimation protocol, post-exercise assessment, training dose, and biopsy depot can influence whether such changes are detected. These findings suggest that thermogenic responses are conditional rather than absent, supporting the depot-, phenotype-, and protocol-specific interpretation adopted below.

In obese older adults characterized by brown fat resistance, exercise is best conceptualized as a multi target intervention (114–116). Resistance training and combined training support preservation of skeletal muscle mass and may influence metabolic regulation through exercise evoked myogenic signals, including transient elevations in irisin and IL 6 (117–119). Aerobic training improves endothelial function and insulin sensitivity (120, 121), thereby facilitating substrate delivery to thermogenic depots, and sustained training can reduce inflammatory inhibition of thermogenic gene programs (122, 123). Accordingly, even when imaging derived indices of BAT show minimal change, as discussed in Box 1, exercise based programs may still yield meaningful improvements in obesity related metabolic health in older populations.

### Endocrine mediators of exercise: myokines and brown adipokines

A key mechanism by which exercise modulates adipose biology involves myokine secretion. During contraction, skeletal muscle releases peptides and metabolites into the circulation that can act on distal targets, including adipose depots (124). Several myokines have been implicated in promoting thermogenic activity.

Myokine irisin: Irisin was first identified as a PGC1-α-dependent myokine generated by proteolytic cleavage of FNDC5 and linked to brown-fat-like development of white adipose tissue (125). In adipocyte studies, irisin has been reported to engage p38 MAPK and ERK signaling, thereby increasing PGC-1α activity and UCP1-related transcription and supporting the browning program in white adipocytes (126). In mouse models, exogenous irisin administration can increase energy expenditure and ameliorate diet induced metabolic dysfunction (127). In humans, studies have reported exercise associated changes in circulating irisin and correlations with imaging derived indices of brown adipose activity (128, 129), but findings are inconsistent across assays, training paradigms, and participant characteristics, highlighting the need for further validation. In obese older adults, particularly those with concomitant sarcopenia, irisin responsiveness may depend strongly on training modality and dose (130). Whether irisin-related signaling translates into durable thermogenic phenotypic changes in obese older adults requires further well-controlled studies.

Interleukin 6 (IL 6): IL 6 is a prototypical exercise responsive cytokine that rises transiently during endurance exercise and is associated with increased lipolysis, greater circulating fatty acid availability, and short term improvements in insulin sensitivity (131). In thermogenic adipose tissue, IL 6 has been implicated in regulation of heat production and systemic metabolic control. In animal models, IL 6 deficiency is associated with impaired cold induced thermogenesis (132), and transplantation studies suggest that adipose derived IL 6 can modulate glucose homeostasis through pathways linked to FGF21 (133). It remains unclear whether exercise-induced IL-6 modifies FGF21 production through intermediary organs (e.g., the liver) and thereby influences brown adipose function.

Meteorin like (METRNL): METRNL is released from skeletal muscle in response to exercise and is also expressed in adipose tissue during cold exposure (134). Preclinical studies indicate that METRNL can improve insulin sensitivity and attenuate inflammation and may facilitate beige adipocyte recruitment through immune mediated adipose remodeling, including shifts toward an anti inflammatory milieu enriched in M2 like macrophage profiles (135). In obese mouse models, increased METRNL expression has been associated with greater white adipose browning and improved metabolic parameters (134). Human investigations have reported associations between circulating METRNL and markers linked to thermogenic adipose activity (136), and some studies observe higher levels after endurance training (137). In obese older adults, the durability of METRNL-related signaling is likely shaped by inflammatory status and training dose, which may influence the persistence of beige-like phenotypes.

Fibroblast growth factor 21 (FGF21): FGF21 is produced by multiple organs and contributes to systemic metabolic regulation, with circulating concentrations influenced by exercise and cold exposure. It has been proposed to facilitate white adipose browning, increase UCP1 related transcription, and enhance fatty acid oxidative capacity (138–140). Although higher basal FGF21 levels are frequently observed in older adults, this does not necessarily indicate increased pathway signaling, and several studies suggest that stimulus responsiveness may be altered in a manner that depends on the underlying metabolic phenotype (141–143). Exercise associated FGF21 signaling may support adaptations relevant to browning resistance by improving substrate handling and modulating catecholamine sensitivity. Whether older adults with obesity exhibit functional FGF21 resistance, and how this axis responds to exercise based interventions, remains a key question for well-characterized elderly cohorts.

Neuregulin 4 (NRG4): Unlike the mediators described above, NRG4 is not a myokine but a brown adipokine — an epidermal growth factor (EGF)-family ligand that is highly enriched in BAT, is further induced during brown adipocyte differentiation and cold-driven recruitment of beige adipocytes in white adipose tissue, and signals principally through ErbB4/ErbB3 receptors on the liver and other peripheral targets (144). Gain- and loss-of-function studies in rodents established that NRG4 attenuates hepatic *de novo* lipogenesis via suppression of SREBP-1c, protects against diet-induced insulin resistance and hepatic steatosis, and supports a healthier systemic adipokine profile alongside enhanced fuel oxidation (144, 145). Adipose expression and circulating concentrations of NRG4 are consistently reduced in rodent and human obesity, in metabolic syndrome, and with advancing age, suggesting that circulating NRG4 may track the functional status of thermogenic adipose tissue (146). Although NRG4 originates from BAT rather than contracting muscle, it responds to exercise training: in a 12-week randomized trial in men with obesity, high-intensity interval training and circuit resistance training elicited greater increases in serum NRG4 than moderate-intensity continuous training, alongside parallel gains in body composition and cardiometabolic risk factors (147). This exercise responsiveness is biologically consistent with evidence that structured training can partially restore BAT functionality and positions NRG4 as a candidate circulating read-out of brown-fat–liver crosstalk in obese older adults. Human observational data nonetheless remain heterogeneous, with some clinical studies reporting discordant associations between circulating NRG4 and insulin sensitivity, so translation to aging populations will require confirmation with prespecified endpoints and standardized assays (146).

Collectively, these myogenic mediators together with the brown adipokine NRG4 support endocrine communication between skeletal muscle, thermogenic adipose depots, and the liver and represent a major route by which exercise modulates lipid metabolism and thermogenic capacity. Repeated training may partially offset sarcopenia related deficits in inter tissue signaling and improve the systemic milieu that enables adipose metabolic adaptation.

Myokine and adipokine signaling patterns are strongly shaped by age and metabolic status (148). Aging is associated with lower PGC-1α expression and a blunted FNDC5/irisin response, and chronic inflammation and insulin resistance may further impair adipose responsiveness to myokine signaling. Findings are also shaped by methodological factors, including sampling time, assay specificity, fasting state, circadian timing, and concurrent medication use. Accordingly, the inconsistent results reported in obese older adults likely reflect phenotype-dependent biology superimposed on measurement variability rather than a true absence of exercise-responsive signaling.

Several investigations propose that exercise associated signaling contributes to initiation or persistence of thermogenic transcriptional programs within adipose tissue (149–151). Human evidence is mainly derived from observational designs and surrogate outcomes, so causal interpretation should remain cautious, especially in obese older adults. Correlational signals should be presented as hypotheses that require confirmation with standardized endpoints and longitudinal designs. Thermogenic adipose tissue may also release mediators that support bidirectional regulation between fat and other organs. For example, experimental studies associate brown adipose derived interleukin 6 with improved systemic glucose homeostasis (152–154). In addition, 12,13-dihydroxy-9Z-octadecenoic acid (12,13-diHOME), an oxylipin derived from linoleic acid and released by BAT in response to cold exposure or exercise, has been reported to increase fatty acid uptake into BAT and skeletal muscle (155). Together, these observations support the possibility of coordinated cross-tissue signaling between skeletal muscle and thermogenic adipose depots.

### Effects of different exercise modalities

Aerobic exercise and resistance training may affect brown and beige adipose function through partially distinct mechanisms, and in applied settings a combined program is often more appropriate. Aerobic training commonly improves cardiorespiratory performance and insulin sensitivity and can enhance substrate handling, thereby creating systemic conditions that may facilitate recruitment of thermogenic adipose depots (156). The magnitude of exercise evoked myokine signaling varies by age and metabolic phenotype.

In rodent models, endurance training can attenuate high fat diet induced metabolic dysfunction and, in some settings, has been reported to increase the abundance of adipocyte precursor populations within brown adipose tissue (157). These observations raise the possibility that endurance exercise influences adipose plasticity and cellular reserve, with human studies showing variable signals depending on study design and endpoint selection. Findings across animal studies are not uniform. Some report increased UCP1 related expression in subcutaneous adipose tissue, whereas others observe minimal change, with discrepancies plausibly attributable to differences in ambient temperature, training load, and species or strain characteristics (158, 159).

Human studies suggest that moderate-intensity aerobic exercise alone does not consistently produce sustained increases in FDG-PET-derived indices of brown adipose activity (160). In a large randomized trial, 24 weeks of combined endurance and resistance training improved cardiorespiratory fitness and body composition in young participants, while no measurable changes were detected in brown fat volume or glucose uptake under the specific endpoints and conditions used (161). One frequently cited interpretation is that exercise induced heat production and higher energy expenditure may transiently reduce the need for non-shivering thermogenesis, such that imaging signals may not fully reflect functional adaptations (162). Because this trial enrolled young, metabolically healthy individuals, response patterns may differ in older adults or in those with cardiometabolic impairment. More broadly, the discrepancy between null imaging findings and positive preclinical data likely reflects the interaction of cohort phenotype, intervention dose, and environmental conditions. Rodent studies are typically conducted in young animals below thermoneutrality, where exercise can readily augment BAT browning, whereas human trials are usually performed near thermoneutrality, where this effect is less readily detected. In obese older adults, low baseline BAT detectability, a higher effective thermoneutral zone, and common use of β-adrenergic dampening medications may further blunt imaging-detectable activation. As a result, phenotypically heterogeneous older cohorts are likely to show variable group-level effects even when individual responders are present.

For obese older adults, aerobic training may be more likely to enhance thermogenic adipose performance through indirect mechanisms, including reductions in visceral adiposity, improved insulin sensitivity, and better tissue perfusion that supports substrate delivery. Therefore, even if its acute mobilizing effect is weaker than cold exposure, sustained training can optimize the systemic milieu and allow residual brown or beige adipose tissue to operate closer to its functional capacity. Divergent conclusions across studies are often attributable to differences in endpoint properties and experimental conditions.

Resistance training primarily promotes preservation and accrual of skeletal muscle mass. By mitigating sarcopenia, it can enhance metabolic reserve and may support production and amplification of exercise responsive myogenic mediators (163). Available data also suggest that resistance exercise can influence anabolic endocrine profiles, potentially contributing to a more permissive hormonal milieu (164).

Evidence linking resistance training to BAT imaging or functional thermogenic outcomes is mixed, and most studies have not focused on obese older adults. High intensity resistance sessions can induce transient catecholamine elevations, which could in principle participate in thermogenic recruitment, but implementation in older adults must be guided by safety considerations and dose feasibility (165, 166). For this population, the most established clinical value of resistance training is maintenance or increase of lean mass, support of resting metabolic rate, and indirect facilitation of thermogenic adipose function through improved substrate handling and a more favorable systemic metabolic environment.

Combined exercise regimens, particularly programs integrating aerobic and resistance components, are widely implemented to improve cardiorespiratory capacity and muscular strength concurrently. Conceptually, this approach is well suited to the multi domain needs of obesity in later life, as it can enhance vascular function and insulin sensitivity while preserving or increasing skeletal muscle mass.

Preclinical data further suggest that pairing exercise with adjunct interventions may augment browning related phenotypes (167). In some animal models, polyphenol supplements paired with exercise training have been linked to larger increases in browning markers in white adipose tissue than either approach on its own (168). This pattern supports the idea of testing combined strategies in human studies. For now, these results should be framed as working hypotheses and research priorities, not as firm conclusions that can be applied directly to people.

When prescribing exercise for obese older adults, safety and a plan that fits the individual matter most. Frail people, and those with heart or blood vessel problems, may not cope well with high intensity exercise or strong cold exposure. A practical starting plan can include two to three light to moderate resistance sessions each week that work the main muscle groups, along with moderate aerobic activity such as brisk walking or water based exercise. A person can increase the load and time step by step as physical capacity improves.

The main goal is to protect muscle and heart health through long term adherence, which can improve whole body conditions that support thermogenic responses. Exercise can improve blood glucose control, lower inflammation, and support blood vessel structure and function.

Across available exercise studies, heterogeneity in training dose, baseline adipose detectability, and endpoint selection remains a key barrier to synthesis in obese older adults. Future trials should incorporate consistent thermogenic outcome definitions, control for temperature and recent activity, and evaluate functional and cardiometabolic endpoints alongside thermogenic surrogates to improve interpretability and translational value. Human evidence in obese older adults remains limited in quantity, and the available studies often involve small sample sizes, with substantial heterogeneity in temperature settings, direct assessments of thermogenic function, and clinically relevant outcome measures. Future studies should plan subgroup analyses in advance, so researchers can look at sex, menopausal status, medication use, baseline detectability of thermogenic adipose tissue, and the presence of sarcopenic obesity. This approach can help identify who is more likely to respond and can guide more suitable exercise plans. For individuals unable to achieve sufficient training volume, pharmacological options or exercise-mimetic approaches may be considered, with implementation best guided by structured safety monitoring and phenotype-informed stratification.

Taken together, the literature suggests that the apparent inconsistency in exercise induced thermogenic responses largely reflects three factors: age related biology, metabolic status, and methodological variability. Reduced sympathetic signaling, β adrenergic desensitization, lower myokine secretion, and declining mitochondrial and progenitor cell reserve may limit responsiveness, while chronic inflammation, insulin resistance, and ectopic lipid accumulation further constrain thermogenic programs. At the same time, differences in training dose, cold exposure and imaging protocols, assay specificity, adipose depot, and measurement timing influence whether such changes are detected. This framework may help distinguish true non-response from design related failure to detect an existing effect and improve cross study synthesis in obese older adults.

## Nutritional factors and thermogenic adipose tissue

Direct evidence for nutrition-driven activation of thermogenic adipose tissue in obese older adults is scarce; therefore, this section emphasizes mechanistic plausibility and translational relevance, while distinguishing these from confirmed intervention effects in older populations. Dietary patterns and nutrient exposures can influence energy expenditure and adipose tissue biology. Beyond total energy intake, selected foods and bioactive constituents may augment thermogenic responses by engaging pathways related to brown adipose tissue activity or by facilitating browning within white adipose depots. Because nutritional strategies can be implemented alongside exercise and are generally more sustainable over extended periods, this section frames them as adjunct modules that support thermogenic phenotypes rather than as primary substitutes for cold exposure or pharmacological stimulation.

The following sections synthesize principal categories of thermogenesis supportive nutrients, outline their putative mechanisms, and integrate findings from experimental models and human studies, with a conceptual overview provided in Figure 4.

Rather than discussing each compound individually, we group candidate nutrients according to their predominant mechanism of action in thermogenic adipose tissue. Four broad categories can be identified: (i) activators of sympathetic and TRP channel signaling that engage the upstream neural input to BAT; (ii) modulators of mitochondrial biogenesis and UCP1 transcription acting through the AMPK-SIRT1-PGC-1α axis; (iii) lipid-derived mediators and substrates that influence inter-organ fuel handling; and (iv) nuclear receptor ligands and enzymatic cofactors that establish a permissive metabolic baseline. This framework helps clarify which compounds act on the same node and are therefore unlikely to produce additive effects, as well as which act upstream or downstream, a distinction that is particularly relevant in obese older adults, in whom upstream sympathetic drive and mitochondrial reserve are already compromised. Table 1 outlines the corresponding preclinical and clinical evidence for representative compounds in each category, together with the recommendations for obese older adults.

### Activators of sympathetic and TRP channel signaling

Caffeine is common in coffee, tea, and many commercial drinks, and it blocks adenosine receptors while also increasing sympathetic signaling (169, 170). Short term studies usually show small rises in resting energy use and modest increases in supraclavicular skin temperature (171). Some experimental work also suggests that regular intake, especially when paired with tea catechins, may relate to higher cold induced thermogenic readouts, even though study designs and outcome measures differ widely across reports (172). Older adults still need careful safety checks, since caffeine can disrupt sleep, worsen anxiety symptoms, and raise blood pressure.

Capsaicin is the pungent compound in chili peppers, and it activates transient receptor potential vanilloid 1 (TRPV1) channels while also engaging sympathetic related signaling (173, 174). Human studies often report small increases in energy use or modest rises in cold related thermogenic readouts after capsaicin exposure, which suggests a possible effect, although the overall size is usually limited (175). A recent systematic review and meta-analysis of randomized trials in overweight or obese adults reported only modest average effects on BMI, body weight, and waist circumference, and the trials varied widely in design and dosing (176). Other pungent and aromatic compounds, such as cinnamaldehyde and gingerol, have been linked in some short term studies to small shifts in energy use or substrate oxidation (177, 178).

### Modulators of mitochondrial biogenesis and UCP1 transcription

Polyphenols are abundant in plant based foods, including fruits, vegetables, tea, and wine, and are commonly linked to antioxidant and anti-inflammatory properties (179). Emerging evidence indicates that selected polyphenols and other phytochemicals may support BAT related phenotypes or facilitate white adipose browning indirectly by modulating mitochondrial adaptation, sympathetic signaling pathways, or adipocyte transcriptional programs (180).

Resveratrol, a polyphenol abundant in grapes and red wine, is among the most intensively investigated dietary bioactives. In multiple rodent studies, resveratrol supplementation has been associated with increased adipose oxidative capacity and upregulation of UCP1 related thermogenic pathways (181). Enhanced expression of thermogenesis associated genes has also been reported in neonatal or early life intervention paradigms (182); however, these developmental models may not translate directly to obesity in later life and should be interpreted cautiously.

In adult obese mice, resveratrol administration has been linked to higher oxygen consumption, increased UCP1 expression in brown adipose tissue, and improvements in glucose metabolic phenotypes, with similar observations reported in models of more severe metabolic disruption (183, 184). Mechanistically, resveratrol is proposed to engage SIRT1 and AMPK signaling, thereby promoting PGC 1α dependent mitochondrial biogenesis and oxidative metabolism programs that align with UCP1 related thermogenic phenotypic shifts (185, 186).

In humans, replication has been variable across trials, likely reflecting differences in dosing, bioavailability, baseline phenotype, and endpoints. In some trials involving obese men, prolonged high dose resveratrol supplementation increased expression of thermogenic markers in subcutaneous adipose tissue, including UCP1, PRDM16, and PGC 1α, together with changes consistent with SIRT1 pathway engagement (187). In contrast, other studies have not detected meaningful reductions in body weight or clear improvements in metabolic outcomes.

Resveratrol has also been proposed to modulate energy balance through effects on the gut microbiome and bile acid signaling (188), although direct supportive evidence in humans remains relatively limited. In older adults, key constraints relate primarily to limited bioavailability and long term tolerability. Accordingly, rather than advocating routine supplementation, a more informative research direction is development of formulations or structural analogs with improved systemic exposure, followed by evaluation using reproducible mechanistic and clinical endpoints in metabolically compromised elderly populations.

Tea catechins, particularly epigallocatechin gallate (EGCG) from green tea, are also widely studied polyphenols. Evidence from intervention studies suggests that catechin rich preparations may increase fat oxidation and produce small increases in energy expenditure, which has motivated their evaluation in weight management (189). In some reports, co administration of EGCG with caffeine has been associated with greater cold related thermogenic readouts, consistent with possible interaction at the level of sympathetic signaling (190, 191), although effect sizes are heterogeneous across studies.

Curcumin, the principal polyphenolic constituent of turmeric, has been investigated for potential links to thermogenic phenotypes. In animal studies, curcumin supplementation has been associated with higher UCP1 expression and increased browning markers in white adipose tissue, accompanied by reduced adiposity and improved insulin sensitivity (192, 193). Proposed mechanisms include activation of AMPK related signaling and dampening of inflammatory pathways.

From a clinical perspective, low bioavailability remains a major limitation, and the evidence that curcumin directly improves human BAT function or produces sustained increases in thermogenic output remains preliminary. Some small clinical trials report added improvements in body weight or glycemic measures (194, 195), yet studies with direct and repeatable human endpoints are still needed to confirm any thermogenic benefit. New formulations that improve solubility, or the use of absorption enhancers taken together with curcumin, may raise systemic exposure, but stronger evidence is needed to define their real clinical value (196).

### Lipid-derived signaling and substrate supply

Dietary fat composition can influence substrate handling and signaling within thermogenic adipose depots. Relative to saturated fatty acids, monounsaturated and polyunsaturated fatty acids are more often examined for associations with brown and beige adipose phenotypes (197). In practice, improving fat quality is more consistently linked to favorable cardiometabolic profiles, including better lipid parameters, reduced inflammatory tone, and enhanced insulin sensitivity, and may therefore provide a metabolic foundation that supports thermogenic responsiveness (198).

Long chain omega 3 polyunsaturated fatty acids, particularly eicosapentaenoic acid (EPA) and docosahexaenoic acid (DHA) from fish oil, are the most frequently discussed examples. In animal models, omega 3 supplementation is often accompanied by increased expression of UCP1 related markers in brown adipose tissue or subcutaneous white adipose tissue and by upregulation of mitochondrial oxidative pathways (199). Mechanistic studies further propose that omega 3 fatty acids can modulate adipocyte signaling through fatty acid receptor mediated pathways and, in some contexts, influence sympathetic input to adipose tissue, with transient receptor potential channel related mechanisms also suggested (200, 201).

Across population level studies, the most consistent effects of omega 3 supplementation are observed in cardiometabolic biomarkers, including reductions in triglycerides and changes in inflammation related measures. Some reports further suggest that fish oil may increase postprandial energy expenditure or related metabolic readouts, potentially reflecting enhanced substrate mobilization (202), although findings are not uniform.

Beyond omega 3 fatty acids, oleic acid is also of interest. Oleic acid is a monounsaturated fat found in high amounts in olive oil. Researchers often interpret these findings as a shift toward more oxidative metabolic programming in fat cells when diets high in oleic acid replace diets high in saturated fat (203). Proposed mechanisms include changes in lipid handling through nuclear receptor driven gene activity, together with more efficient β oxidation that may provide fuel for uncoupling linked energy dissipation.

Omega 3 fatty acids have stronger evidence for lowering triglycerides (204). From an inter organ communication perspective, thermogenic adipose tissue both generates and responds to lipid derived mediators. A prominent example is 12,13 diHOME, an oxidized linoleic acid metabolite that has been linked to brown adipose activation during cold exposure. Experimental and translational findings suggest that 12,13 diHOME may enhance fatty acid uptake by skeletal muscle, thereby coordinating substrate utilization between brown adipose tissue and muscle (205).

Interindividual variation in circulating 12,13-diHOME appears substantial across human studies, and observed associations may depend on baseline metabolic state and study design, including temperature exposure and sampling conditions. In this context, dietary linoleic acid is best interpreted as a background substrate rather than a direct lever for increasing 12,13-diHOME in a predictable manner. A more appropriate approach is to maintain a balanced intake of essential fatty acids and to avoid treating single fatty acids or their metabolites as actionable standalone intervention targets.

### Nuclear receptor-mediated transcriptional programming: Vitamins A and D

Vitamins A and D exert their effects through the ligand-activated nuclear receptors RAR and VDR, which are involved in adipocyte differentiation and the transcriptional regulation of oxidative and thermogenic programs. Because the strength of these signals depends on receptor occupancy rather than simply on nutrient intake, supraphysiological exposure does not consistently translate into greater thermogenic effects. In clinical practice, the most meaningful application of both vitamins remains the correction of deficiency.

Vitamin A and its metabolites, particularly retinoic acid, are closely linked to adipocyte differentiation, adipose tissue metabolism, and thermogenic programming, as supported by mechanistic studies in mice and analyses of human adipose tissue retinoic acid biosynthesis (206, 207). Experimental work shows that retinoic acid signaling can change oxidative metabolism and thermogenesis related gene programs under certain conditions, as seen in mouse studies where adipocyte retinoic acid receptor α signaling increased energy use and reduced metabolic injury (208). These effects depend strongly on dose and setting, making it hard to translate them into lasting clinical benefit in obese older adults, even though a recent mouse study reported that brown fat-specific overexpression of retinol-binding protein 4 (RBP4) can increase cold-induced thermogenesis (209). In addition, preclinical studies have shown that retinoid-based interventions may reduce adiposity and improve obesity-related metabolic phenotypes in mice; for example, all-trans retinoic acid administration has been reported to reduce fat accumulation, and more recent work further showed reduced body weight and white adipose tissue accumulation in obese mice (210, 211).

In clinical practice, vitamin A supplements are best used to correct deficiency. Treatment of low intake or malabsorption can help maintain normal metabolism and tissue health. Many older adults already have enough liver stores, so high dose supplementation without a clear need may raise the risk of adverse events, including hepatotoxicity. A safer approach prioritizes dietary sources of provitamin A carotenoids, such as beta carotene rich vegetables and tubers, and reserves supplements for confirmed deficiency after professional assessment and follow up monitoring.

Vitamin D signaling connects with pathways involved in energy metabolism, and vitamin D insufficiency is common in obese older adults, which has led to interest in possible links with thermogenic adipose phenotypes. Vitamin D receptor signaling has been implicated in adipocyte differentiation, lipogenesis, and inflammatory responses (212), and mechanistic work has further shown that vitamin D can regulate fatty acid composition in subcutaneous adipose tissue through Elovl3 (213). The evidence base still comes mainly from mechanistic work and animal models; human studies vary in design, dosing, and endpoint selection (Box 1). Recent human work also reported no clear association between circulating 25-hydroxyvitamin D and BAT metabolism in healthy adults (214).

Clinicians generally view vitamin D as a basic part of nutritional care in older adults. Sunlight exposure, diet, and supplements can correct deficiency and support skeletal and immune health, and these effects may help metabolic flexibility in a broader way. Evidence for thermogenesis remains limited, and current human data do not consistently show that routine vitamin D supplementation at standard doses produces lasting increases in BAT thermogenic function in adults. At the same time, disease-context-specific animal data suggest that vitamin D repletion can attenuate aberrant adipose browning and thermogenic gene expression, as shown in a mouse model of CKD-associated cachexia (215). Animal studies still report depot specific and context dependent thermogenic changes when vitamin D status is altered in rats and mice (216, 217). Vitamin D should therefore not be framed as a main thermogenic intervention.

In older adults, supplementation should focus on correcting deficiency and should match the individual clinical setting, including renal function, prior hypercalcemia, nephrolithiasis risk, and current medications. Clinicians can monitor serum calcium and 25-hydroxyvitamin D when clinical circumstances make it necessary.

### Micronutrient cofactors for mitochondrial and oxidative metabolism: B vitamins, choline, iron, and zinc

B vitamins, choline, iron, and zinc all serve as essential cofactors in one-carbon metabolism, the electron transport chain, and redox enzymes that support the oxidative capacity required for UCP1-mediated thermogenesis. Their role is therefore better understood as permissive rather than stimulatory. Adequate levels help avoid a metabolic ceiling, whereas supraphysiological dosing does not appear to provide additional thermogenic drive and may instead introduce off-target risks, including zinc-induced copper deficiency and iron overload-related dysmetabolism. In obese older adults, the more appropriate clinical focus is to identify and correct deficiency rather than to use supplementation as a means of enhancing thermogenesis.

B vitamins and choline play key roles in cellular energy use and one carbon metabolism, and they act as cofactors that shape substrate handling and mitochondrial processes. *In vitro* studies suggest that some components can influence beige related features under specific experimental settings and within narrow dose ranges (218), and recent work supports this by showing that thiamine availability can affect thermogenic activation in human adipocytes (219). Beyond adipocyte experiments, thiamine deficiency has also been reported in individuals with obesity and in bariatric surgery candidates, including preoperative cohorts and medically complicated obesity populations (220–223). These findings support careful perioperative assessment and correction of thiamine deficiency in the bariatric setting rather than assuming adequate thiamine status. Recent human adipose studies further indicate that SLC19A3, which encodes thiamine transporter 2, shows adipose-enriched expression, increases during adipocyte differentiation, and is detectable in both brown and white adipose depots, strengthening the relevance of thiamine handling for adipocyte function (224). These findings still depend heavily on experimental context, so they do not support high dose supplementation as a way to boost thermogenesis.

In older adults, clinical care should focus on finding and correcting nutritional deficiencies. Low plasma thiamine concentrations have been reported in type 1 and type 2 diabetes (225), and high-dose thiamine supplementation has been reported to improve glucose tolerance in hyperglycemic individuals (226). In addition, case reports of Wernicke’s encephalopathy have described severe hypothermia responding to thiamine treatment (227), underscoring the systemic consequences of marked deficiency. Vitamin B_12_ insufficiency and malabsorption are relatively common, and many people may also have low habitual choline intake. Balanced dietary patterns, or a suitable multivitamin chosen through clinical assessment, can help close these gaps and support overall metabolic health. People with multiple comorbidities and polypharmacy also need individualized safety planning. Periodic assessment of vitamin B_12_ status may be appropriate in long term users of metformin or proton pump inhibitors.

Iron is required for many parts of the mitochondrial electron transport chain. Iron supports energy metabolism by enabling ATP production and normal electron transport chain function (228). Disrupted iron homeostasis, marked by high serum ferritin and excess iron build up in the liver, adipose tissue, and skeletal muscle, can worsen adipose dysfunction (229, 230). Mitochondrial biogenesis and function also rely on iron. Experimental studies show that excess iron in adipose tissue during browning, induced by β3 AR activation, is linked to higher mitochondrial formation and respiration (231), and recent mouse work also suggests that iron supplementation can promote adipocyte thermogenesis through lipolysis related pathways (232). Mouse studies also show that altering adipose iron handling can raise mitochondrial respiration and adipokine expression in BAT (233), and newer ferritin heavy chain knockout work has reported improved adaptive thermogenesis and better metabolic profiles (234). Iron deficiency can impair beige fat formation, while iron chelation can enhance beige fat differentiation and metabolic activity.

Human evidence should, however, be interpreted with caution. In human adipose tissue, TFRC expression has been reported to increase in browning fat but to decrease in adipose tissue from overweight individuals. TFRC was also negatively correlated with body mass index and positively correlated with UCP1, suggesting that reduced TFRC may be associated with an impaired thermogenic phenotype in obesity rather than higher TFRC levels in overweight individuals *per se* (235). More recent work further showed that adrenergic stimulation increased TFRC expression in human brown adipocytes and that transferrin was constitutively secreted by these cells, supporting a role for iron handling during thermogenic activation (236). Together, these findings support the biological plausibility of iron related regulation in beige adipocyte development, with phenotype specific effects likely across aging and obesity.

Zinc is an essential trace element that supports many enzymes and is closely tied to immune function and nervous system stability. Zinc status may shape metabolic adaptation in an indirect way because it supports tissue repair and the broader neuroimmune environment. In older adults, supplementation should mainly target deficiency correction, and large scale clinical data still show that zinc deficiency becomes more common with age (237, 238). Mild insufficiency is relatively common and can often be improved through foods such as nuts, legumes, and seafood. Supplements should be reserved for confirmed deficiency and used to restore levels back to the normal physiological range, since animal studies also suggest that zinc exposure can affect adipose lipid handling and inflammation in ways that may not always be beneficial. Excess zinc can also reduce copper absorption and cause secondary copper deficiency, so dosing should aim for adequacy rather than high exposure, and recent clinical reports continue to describe zinc induced copper deficiency in older adults (239).

Nutritional studies relevant to thermogenic adipose responses often vary in dosing, formulation, intervention duration, and concurrent lifestyle context, which complicates attribution to single nutrients. Future work should focus on pragmatic dietary patterns, combine reproducible biomarkers with interpretable thermogenic endpoints, and evaluate feasibility and safety in older adults with common comorbidities and medication exposure.

### Synergistic interventions and clinical implications

For obese older adults, combined endurance and resistance training should form the foundation of intervention, as skeletal muscle contraction promotes exercise-responsive mediators, including irisin, IL-6, FGF21, and METRNL, while reducing adverse myostatin signaling, thereby helping shape the endocrine and paracrine milieu that supports browning signals and BAT responsiveness. We suggest moderate-intensity aerobic training at 40% to 60% VO2 reserve, or an RPE of 12–14, for 150–300 min per week, together with progressive resistance training 2–3 times per week involving major muscle groups and 8–12 repetitions per set. This combination addresses both metabolic flexibility and preservation of muscle mass. Exercise prescriptions should be individualized according to comorbidity profile. Low-impact modalities such as aquatic exercise or recumbent cycling may be more suitable for individuals with osteoarthritis or balance impairment. In those with cardiovascular disease, intensity should be advanced cautiously after appropriate pre-participation screening. Supervised initiation may be particularly appropriate in the setting of frailty, recent hospitalization, or insulin-treated diabetes. Adherence may be improved by using shorter, more frequent sessions and home-based options, given the mobility and transportation barriers commonly faced by this population. Figure 5 summarizes this integrated framework, including the principal exercise- and nutrition-related inputs and the barriers most likely to constrain net effect.

Dietary optimization can complement these effects by influencing both substrate availability and pathway-specific signaling. Adequate protein intake, at 1.0–1.2 g/kg/day and increasing to 1.2–1.5 g/kg/day when combined with resistance training, distributed across meals at about 25–30 g per meal, helps support muscle protein synthesis and may counteract sarcopenia-related reductions in exerkine production. A Mediterranean-style dietary pattern provides omega-3 PUFAs, with at least 250 mg/day of EPA plus DHA as a general nutritional target rather than a BAT-specific dose; these lipids may support cardiometabolic health and lipid-mediated signaling relevant to thermogenic tone, while MUFAs offer additional substrate and signaling support. Adjunctive thermogenic nutrition strategies may also include moderate caffeine or tea catechin intake to support sympathetic BAT activation, culinary spices such as capsaicin, cinnamaldehyde, and ginger that engage TRPV1- or TRPA1-related browning pathways, and polyphenols including resveratrol and curcumin, which may promote mitochondrial biogenesis and UCP1-permissive signaling through AMPK and SIRT1. Human effect sizes remain modest and appear to depend on context. Caution is needed in the setting of chronic kidney disease, where protein intake may require adjustment, as well as in patients using anticoagulants, those with arrhythmia or uncontrolled hypertension, and those receiving multiple drugs with narrow therapeutic indices.

Clinical benefit ultimately depends on whether three principal barriers can be at least partly addressed: sarcopenia, which reduces exerkine production; inflammaging, in which elevated TNF-α and IL-1β impair mitochondrial function and suppress UCP1; and β-adrenergic resistance, which limits sympathetic signaling to BAT. In selected patients, lifestyle intervention may be combined with pharmacotherapy. GLP-1 receptor agonists can support clinically meaningful weight loss, but concurrent resistance training and adequate protein intake are needed to reduce accompanying lean-mass loss, whereas β3-adrenergic agonists remain investigational. Variation in response is likely related to baseline metabolic phenotype, sex, body composition, medication burden, and inflammaging status. Biomarker-guided stratification using circulating mediators such as 12,13-diHOME, FGF21, and irisin, together with functional and metabolic endpoints rather than weight or imaging alone, may help identify those most likely to respond. When these barriers are at least partly overcome, the overall effects may include greater BAT and beige activation, improved glucose, lipid, and blood pressure profiles, and clinically meaningful benefit even in the absence of substantial weight loss.

## Conclusion

Late-life obesity is accompanied by an age-related decline in the recruitable capacity of brown and beige adipose tissue, arising from impaired sympathetic signaling, chronic low-grade inflammation, endocrine remodeling, and weaker muscle-derived cues. Combined exercise and nutritional strategies remain the most defensible basis for clinical management, and their metabolic and functional benefits, including improved glucose disposal, lipid clearance, and lower cardiometabolic risk, may still be clinically meaningful even when weight loss is modest. Intervention assessment should therefore give priority to metabolic and functional outcomes rather than weight change alone.

Several important gaps remain. First, adequately powered trials in obese adults aged 65 years and older are still scarce, and much of the current evidence is extrapolated from younger or non-obese populations. Second, standardized definitions of thermogenic adipose tissue activation that integrate histological, transcriptomic, and imaging-based criteria are still lacking, which continues to contribute to inconsistent findings. Third, predictors of response and tools for stratification remain underdeveloped, and key questions about who benefits, to what extent, and through which mechanisms have yet to be answered. Fourth, the relative contributions of UCP1-dependent and UCP1-independent thermogenic pathways in older adults, their interaction with sarcopenic obesity, inflammaging, and polypharmacy, and the durability of induced beige adipocyte phenotypes after stimulus withdrawal require further mechanistic and translational investigation.

Future work should therefore focus on four priorities: randomized trials in obese older adults with prespecified subgroup analyses by sex, frailty status, and comorbidity profile; harmonized outcome frameworks linking circulating biomarkers such as 12,13-diHOME, FGF21, and irisin to clinically meaningful endpoints; head-to-head comparisons of exercise and nutrition combinations versus pharmacological adjuncts such as GLP-1 receptor agonists; and safety and adherence data extending to at least 12 months in high-risk subgroups. Until these gaps are addressed, brown and beige adipose tissue should be viewed as a promising but still provisional therapeutic lens in late-life obesity.
