# Supplementary material for: Thermogenic adipose tissue in older adults with obesity: a narrative review of mechanisms, brown fat resistance, and the translational relevance of exercise and nutrition
Source: Front Nutr. 2026 May 14;13:1818342. doi: 10.3389/fnut.2026.1818342 (PMC13216019; doi:10.3389/fnut.2026.1818342)
Supplement: Supplementary file 1 [file Data_Sheet_1.docx]

**Supplementary Material 1**

**Search strategy summary and staged relevance-screening process**

# Overview

This supplementary material summarizes the database-specific search records and staged relevance-screening process used for this structured narrative review. Searches were conducted in PubMed/MEDLINE, Scopus, and Web of Science Core Collection. The search was organized around three predefined Boolean concept combinations: thermogenic adipose tissue-related terms combined with ageing/obesity-related terms, thermogenic adipose tissue-related terms combined with exercise-related terms, and thermogenic adipose tissue-related terms combined with nutrition-related terms. Searches were conducted from database inception to 31 December 2025 and were limited to English-language, peer-reviewed publications.

Records were exported according to the database search-history results and imported into EndNote for deduplication. Because this article was designed as a structured narrative review rather than a systematic review or meta-analysis, study selection was conducted using a staged relevance-screening approach.

The search strategy is reported as database-specific search records and Boolean concept blocks based on the actual retrieval records used for screening. Because the purpose of this structured narrative review was to synthesize mechanistic, translational, and clinical evidence rather than to perform an exhaustive systematic review or meta-analysis, the search strategy is presented as database-specific search records, field settings, Boolean concept blocks, and staged relevance-screening procedures. This format was intended to improve transparency and traceability without implying that the review followed a full systematic-review protocol.

# Eligibility criteria

## Inclusion criteria

1. Studies or reviews focused on brown adipose tissue, beige/brite adipocytes, adipose browning, UCP1-related thermogenesis, or thermogenic adipose tissue regulation.
2. Studies addressing ageing, obesity, exercise, physical activity, diet, nutrition, or metabolic regulation in relation to thermogenic adipose tissue or adipose tissue plasticity.
3. Human studies, animal studies, cell-based mechanistic studies, and key reviews relevant to the scope of this structured narrative review.
4. Studies providing mechanistic, translational, or clinical context for thermogenic adipose tissue in older adults with obesity or related metabolic risk.

## Exclusion criteria

1. Records outside the main scope of brown/beige adipose tissue, adipose browning, UCP1-related thermogenesis, ageing, obesity, exercise, or nutrition.
2. Records with limited mechanistic or translational relevance to the review aims.
3. Records addressing exercise, nutrition, obesity, or metabolic disease without a clear link to thermogenic adipose tissue or adipose tissue plasticity.
4. Records substantially overlapping with more directly relevant or more recent evidence.
5. Publication types or records with insufficient relevance for inclusion in the narrative synthesis.
6. Abstracts, editorials, letters, preprints, duplicate records, studies limited to fetal or neonatal populations, and records for which the full text was not accessible were excluded.

**Table S1. Database-specific search records, search-field settings, and Boolean concept blocks**

| **Database** | **Search block** | **Boolean concept structure** | **Records identified** |
| --- | --- | --- | --- |
| PubMed/MEDLINE | Search 1 | Thermogenic adipose tissue-related terms AND ageing/obesity-related terms | 9,065 |
| PubMed/MEDLINE | Search 2 | Thermogenic adipose tissue-related terms AND exercise-related terms | 3,446 |
| PubMed/MEDLINE | Search 3 | Thermogenic adipose tissue-related terms AND nutrition-related terms | 7,529 |
| PubMed/MEDLINE | Combined search | Search 1 OR Search 2 OR Search 3 | 12,204 |
| Scopus | Search 1 | Thermogenic adipose tissue-related terms AND ageing/obesity-related terms | 11,375 |
| Scopus | Search 2 | Thermogenic adipose tissue-related terms AND exercise-related terms | 5,328 |
| Scopus | Search 3 | Thermogenic adipose tissue-related terms AND nutrition-related terms | 10,187 |
| Scopus | Combined search | Search 1 OR Search 2 OR Search 3 | 15,042 |
| Web of Science Core Collection | Search 1 | Thermogenic adipose tissue-related terms AND ageing/obesity-related terms | 10,346 |
| Web of Science Core Collection | Search 2 | Thermogenic adipose tissue-related terms AND exercise-related terms | 5,205 |
| Web of Science Core Collection | Search 3 | Thermogenic adipose tissue-related terms AND nutrition-related terms | 8,019 |
| Web of Science Core Collection | Combined search | Search 1 OR Search 2 OR Search 3 | 12,600 |

**Note.** The search was organized around three predefined Boolean concept combinations. The three searches within each database were combined using OR. Search fields and syntax were adapted to each database interface, and records were exported according to the database search-history results. Search-field settings were PubMed title/abstract fields, Scopus TITLE-ABS-KEY fields, and Web of Science Core Collection topic fields.

# Staged relevance-screening process

All records retrieved from PubMed/MEDLINE, Scopus, and Web of Science Core Collection were imported into EndNote. Duplicate records were removed before screening. After deduplication, two authors independently screened titles, abstracts, and keywords for relevance to the review topic.

Records clearly unrelated to brown adipose tissue, beige/brite adipocytes, adipose browning, UCP1-related thermogenesis, ageing, obesity, exercise, nutrition, or metabolic regulation were excluded during the initial relevance-screening stage. Potentially relevant records were retained for detailed relevance assessment, including full-text review when necessary. Studies and key reviews were prioritized when they provided mechanistic insight, human translational relevance, or direct evidence related to the review scope.

Records were not prioritized for the narrative synthesis when they were outside the main scope of the review, had limited relevance to the review aims, or overlapped with more directly relevant studies. A total of 208 studies and key reviews were prioritized for the core narrative synthesis, with additional background, methodological, and contextual references cited where appropriate. The figure of n = 208 reflects records identified and prioritized through the systematic search strategy described above; an additional 31 foundational and methodological references (e.g., landmark mechanistic studies, definitional sources, and methodological frameworks such as SANRA) were cited during writing to support background, conceptual, and methodological points, yielding a total of 239 references in the revised manuscript.

**Supplementary Figure 1. PRISMA-style flow diagram**


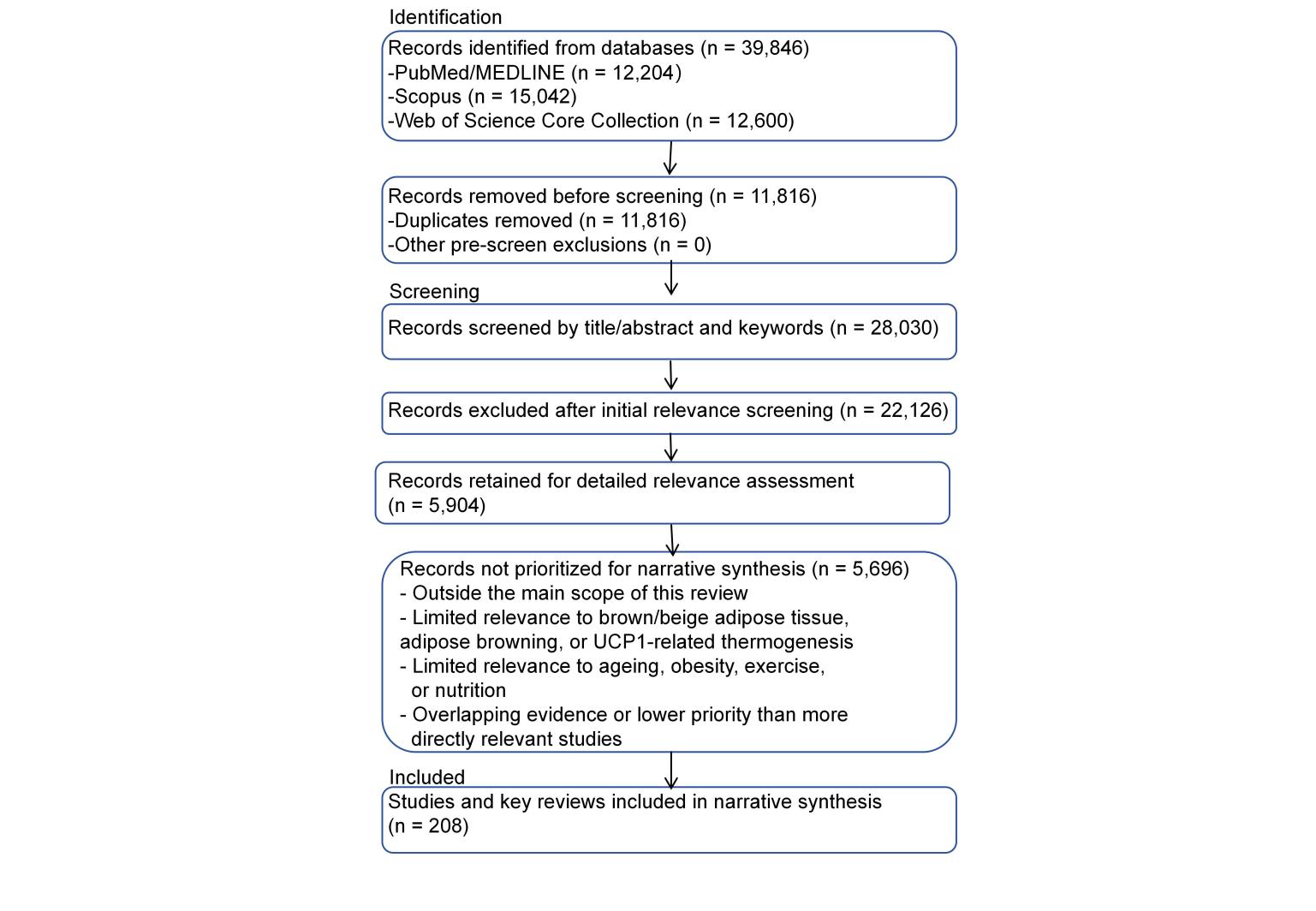


Supplementary Figure 1. PRISMA-style flow diagram of the staged literature search and relevance-screening process for this structured narrative review.
